# Combination of Yaobitong capsules and lumbar oblique pull manipulation for moderate pain in lumbar disc herniation with radiculopathy: a multicenter, randomized, three-arm, parallel-group controlled trial

**DOI:** 10.3389/fneur.2026.1853703

**Published:** 2026-06-26

**Authors:** Guangqi Lu, Minghui Zhuang, Bin Tang, Jirong Zhao, Shaofeng Yang, Jiayi Guo, Ping Wang, Yikai Li, Shaojun Li, Bolai Chen, Puwei Yuan, Hong Jiang, Yusong Jia, Bin Shi, Xuefeng Guan, Yanming Xie, Minshan Feng, Zhefeng Jin, Jinjing Wang, Zelong Zhao, Jiawen Zhan, Xunlu Yin, He Yin, Ming Chen, Kai Sun, Xin Chen, Jie Yu, Liguo Zhu

**Affiliations:** 1 Wangjing Hospital, China Academy of Chinese Medical Sciences, Beijing, China; 2 The First Affiliated Hospital of Guangxi University of Chinese Medicine, Nanning, Guangxi, China; 3 Gansu Provincial Hospital of Traditional Chinese Medicine, Lanzhou, Gansu, China; 4 The First Hospital of Hunan University of Chinese Medicine, Changsha, Hunan, China; 5 Luoyang Orthopedic-Traumatological Hospital of Henan Province, Luoyang, Henan, China; 6 First Teaching Hospital of Tianjin University of Traditional Chinese Medicine, Tianjin, China; 7 Nanfang Hospital, Southern Medical University, Guangzhou, Guangdong, China; 8 Affiliated Hospital of Changchun University of Chinese Medicine, Changchun, Jilin, China; 9 Guangdong Provincial Hospital of Chinese Medicine, Guangzhou, Guangdong, China; 10 Affiliated Hospital of Shaanxi University of Chinese Medicine, Xianyang, Shanxi, China; 11 Suzhou Hospital of Traditional Chinese Medicine, Suzhou, China; 12 Dongzhimen Hospital, Beijing University of Chinese Medicine, Beijing, China; 13 Affiliated Hospital of Shandong First Medical University, Jinan, Shandong, China; 14 Liaoning University of Traditional Chinese Medicine, Shenyang, Liaoning, China; 15 Institute of Basic Research in Clinical Medicine, China Academy of Chinese Medical Sciences, Beijing, China

**Keywords:** lumbar disc herniation with radiculopathy, lumbar oblique pull manipulation, moderate pain, randomized controlled trial, Yaobitong capsules

## Abstract

**Background:**

Lumbar disc herniation with radiculopathy (LDHR) is a leading cause of disability worldwide. For patients with moderate pain, Traditional Chinese Medicine approaches, including Yaobitong (YBT) capsules and lumbar oblique pull manipulation (LOPM), are widely used in clinical practice, often in combination. However, high-quality evidence supporting the superiority of this combination over monotherapy is lacking. This multicenter, randomized, three-arm, parallel-group controlled trial conducted at 13 tertiary hospitals in China aimed to evaluate the efficacy and safety of combining YBT with LOPM in LDHR patients with moderate pain.

**Methods:**

A total of 426 LDHR patients with moderate pain (Visual Analogue Scale [VAS] score ≥ 4 to < 7) were randomly assigned to receive either YBT alone (*n* = 146), LOPM alone (*n* = 137), or a combination of both (*n* = 143). The primary outcome was change in the Oswestry Disability Index (ODI) from baseline to week 2. Secondary outcomes included leg pain VAS scores, low back pain VAS scores, 12-Item Short Form Health Survey (SF-12), adverse events, and disc resorption rates. Outcome measurements were assessed at baseline, 3 days, 1 week, 2 weeks, 6 weeks, 14 weeks, and 26 weeks.

**Results:**

At 2 weeks, the mean reduction in ODI was −19.4 (95% CI, −21.4 to −17.5) in the YBT group, −19.4 (95% CI, −21.4 to −17.5) in the LOPM group, and −21.1 (95% CI, −23.0 to −19.1) in the combination group. The adjusted mean difference was 0.5 (95% CI, −2.3 to 4.9; *p* = 0.99) for YBT versus combination and 1.3 (95% CI, −2.1 to 5.1; *p* = 0.68) for LOPM versus combination. No statistically significant differences in ODI reduction were detected between groups at any follow-up time point (all *p* > 0.05). No statistically significant differences in any of the secondary outcomes were detected between groups at any follow-up time point (all *p* > 0.05). No apparent safety signal was observed in any of the three groups during the short 2-week intervention period.

**Conclusion:**

The combination therapy with YBT and LOPM was not superior to either monotherapy in improving functional disability, pain, or quality of life among LDHR patients with moderate pain.

**Clinical trial registration:**

https://www.chictr.org.cn, ChiCTR2200066051.

## Introduction

Low back pain is the foremost contributor to disability and reduced productivity worldwide (1). In 2020, an estimated 619 million people globally were affected by this condition, with projections indicating that this figure could reach 843 million by 2050 (2). Among the various underlying causes, lumbar disc herniation with radiculopathy (LDHR) represents a major source of disabling low back pain. With an annual incidence affecting 1 to 3% of the population and a peak prevalence among adults aged 30 to 50 years (3), LDHR imposes substantial clinical and socioeconomic burdens. Consequently, developing effective strategies for the prevention and management of LDHR has become an urgent public health priority.

Treatment approaches for LDHR include both surgical and non-surgical modalities, with the selection primarily guided by symptom severity. Surgical intervention is required in certain urgent situations, particularly in cases of cauda equina syndrome (4). While effective, surgical procedures are associated with considerable expenses and inherent risks (5). Conservative management proves adequate for achieving symptom resolution in up to 85% of patients (3), positioning it as the recommended first-line strategy for most individuals, especially true during the first six weeks of care (6, 7).

Traditional Chinese Medicine (TCM) approaches, particularly Chinese patent medicines and manual therapies, have demonstrated promise in the management of LDHR. Evidence indicates that these interventions can enhance patients’quality of life, reduce discomfort, and facilitate the restoration of motor function, while maintaining a favorable safety profile (8–10). Yaobitong (YBT) capsule, a Chinese patent medicine approved by the National Medical Products Administration (NMPA) for treating LDHR, is widely used in orthopedic clinical practice in China (11). Similarly, lumbar oblique pull manipulation (LOPM) is a characteristic manual therapy technique extensively employed in Chinese orthopedic and rehabilitation settings (12, 13). Accumulating evidence over recent years has indicated that the integration of internal and external therapeutic approaches may yield synergistic benefits in the treatment of musculoskeletal disorders (14–16). However, despite the widespread combined use of YBT and LOPM for LDHR in Chinese clinical practice, no randomized controlled trial (RCT) systematically evaluated the efficacy of this combination therapy.

Considering that patients with moderate pain constitute the predominant clinical population with LDHR and given that restricting the study cohort to this specific pain stratum helps minimize bias arising from baseline pain heterogeneity, thereby enhancing the internal validity of the findings, this subgroup was selected as the target population for our trial.

Therefore, we conducted a multicenter, randomized, three-arm parallel-group controlled trial to assess the efficacy and safety of combined YBT and LOPM therapy in LDHR patients with moderate pain.

## Methodology

The trial protocol was approved by the institutional review board of Wangjing Hospital, China Academy of Chinese Medical Sciences (WJEC-KT-2022-026-P002) and is available in Supplementary material 1. Written informed consent was obtained from participants before randomization. This study followed the Consolidated Standards of Reporting Trials (CONSORT) reporting guideline (17).

### Study design

**Figure 1:**
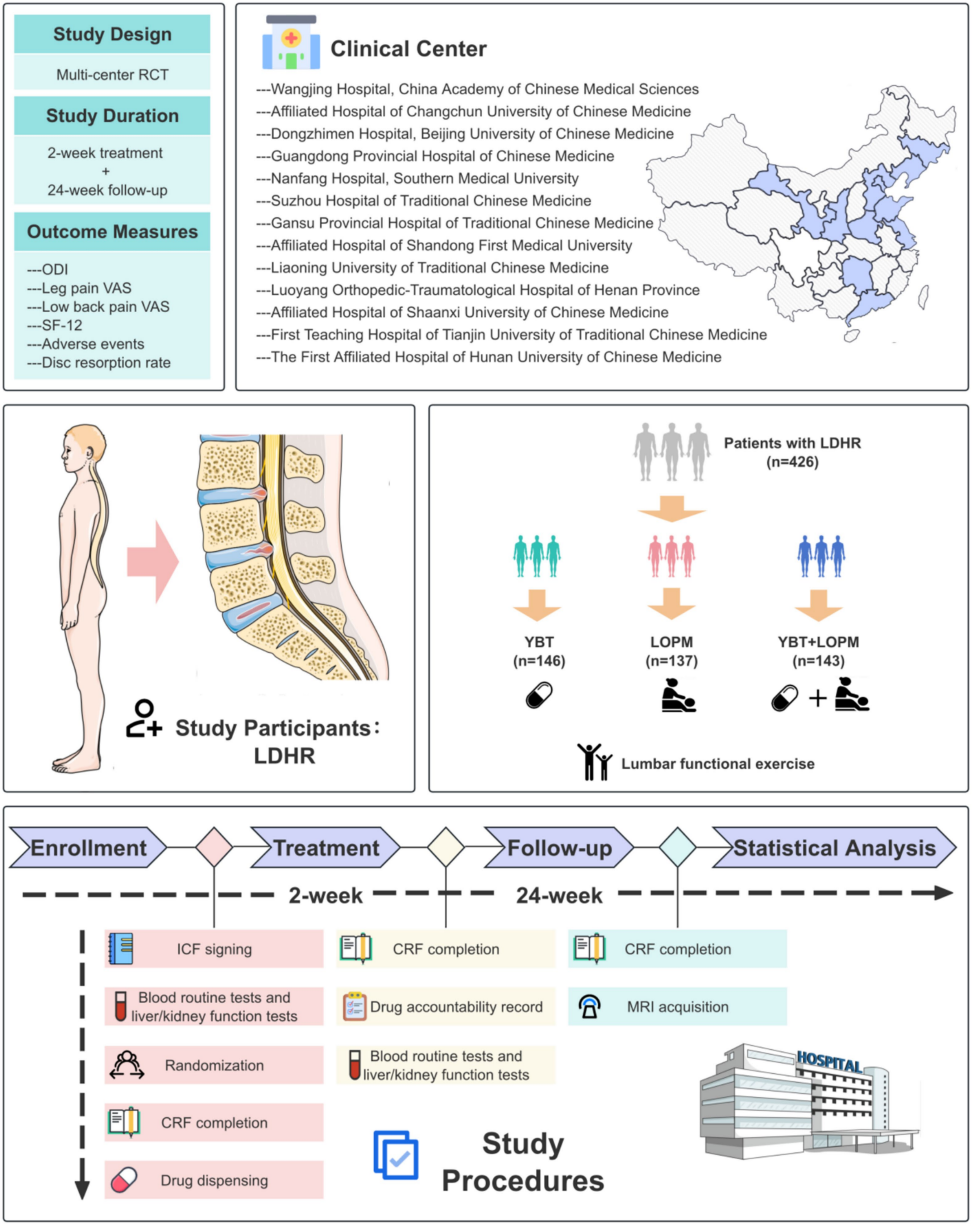
Study flow diagram. CRF, case report form; ICF, informed consent form; LDHR, lumbar disc herniation with radiculopathy; LOPM, lumbar oblique pull manipulation; MRI, magnetic resonance imaging; ODI, Oswestry Disability Index; RCT, randomized controlled trial; SF-12, 12-Item Short Form Health Survey; VAS, visual analog scale; YBT, Yaobitong.

This was a multicenter, randomized, three-arm, parallel-group controlled trial that was conducted at 13 tertiary hospitals in China. Participant recruitment occurred from May 2023 to February 2025 via posters and advertisements on WeChat (a popular social media and messaging application in China). The study flow is in Figure 1.

### Participants

**Figure 2:**
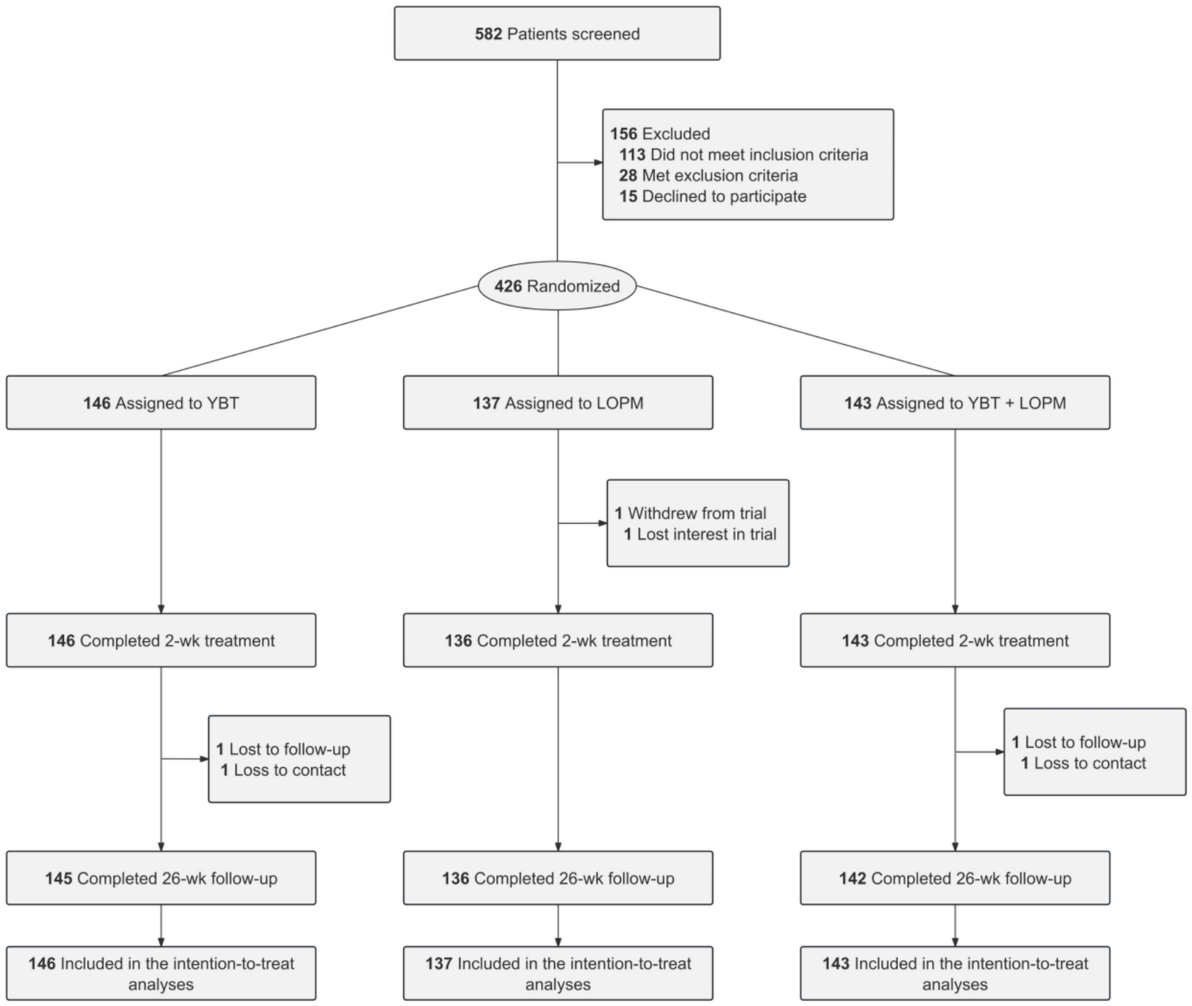
CONSORT participant flow diagram. ITT, intention-to-treat; LOPM, lumbar oblique pull manipulation; YBT, Yaobitong.

Eligible participants were adults aged 18 to 65 years who met the diagnostic criteria for LDHR and reported moderate low back or leg pain. The diagnosis of LDHR was made in accordance with guidelines from the North American Spine Society (NASS) (18) and the Chinese Orthopaedic Association (COA) (19), requiring both (1) imaging evidence (magnetic resonance imaging [MRI] and computed tomography [CT] showing disc herniation with nerve root compression) and (2) correlating clinical symptoms and signs of radiculopathy consistent with the level of nerve root compression shown on imaging (e.g., leg-dominant pain in a dermatomal distribution, with or without sensory, motor, or reflex abnormalities). Moderate low back or leg pain was defined as a score of ≥ 4 and < 7 on Visual Analog Scale (VAS) (0–10 points) (20). Participants were excluded if they had any of the following: (1) History of spinal surgery; (2) Spinal compression fractures; lumbar spondylolisthesis grade II or higher; lumbar spondylolysis; lumbar spinal stenosis; (3) Spinal tumors; spinal tuberculosis; severe osteoporosis (T-score ≤ − 3.0 or associated osteoporotic fractures); diabetes with peripheral neuropathy; (4) Known allergy or hypersensitivity to non-steroidal anti-inflammatory drugs (NSAIDs), Chinese herbal medicines, or other related medications; (5) History of gastrointestinal ulcer or bleeding; (6) Recent coronary artery bypass graft surgery; current use of dual antiplatelet therapy; presence of coagulation disorders or high risk of bleeding; (7) Severe skin diseases or skin lesions in the lumbar region; (8) Pregnancy or breastfeeding; (9) Severe heart failure, stroke, or other major cardiovascular or cerebrovascular diseases; severe hepatic or renal dysfunction; (10) Cauda equina syndrome; lower limb muscle strength of grade 3 or less (on a 0–5 scale); or persistent loss of motor/sensory function with clear indications for surgical intervention; (11) Inability to provide informed consent or comply with study procedures (e.g., significant visual, hearing, or speech impairment; intellectual disability; or severe mental disorder). The full inclusion and exclusion criteria are detailed in the Supplementary material 1. Prior to randomization, all potential participants underwent lumbar spine X-ray, CT, and MRI examinations, as well as the straight leg raise test, to determine their eligibility based on the aforementioned inclusion and exclusion criteria. Between May 2023 to February 2025, we screened 582 patients, of which 426 patients were randomized (Figure 2).

### Randomization and blinding

An independent third party, the Institute of Basic Research in Clinical Medicine at the China Academy of Chinese Medical Sciences, generated the randomization sequence and oversaw the masking procedures. A stratified block randomization method was employed, using study center as stratification factors. Random sequences with a block size of 6 were generated using SAS, version 9.4 (SAS Institute). Allocation concealment was ensured through a password-protected, central online randomization system. Investigators could access the treatment assignment only after a patient had completed eligibility confirmation and provided written informed consent. Outcome assessors and statisticians were all masked to treatment assignment.

### Interventions

Participants were randomly assigned in a 1:1:1 ratio to one of three treatment groups: (1) The YBT group: YBT capsules (Jiangsu Kanion Pharmaceutical Co., Ltd.; National Drug Approval No. Z20010045) were administered orally at a dose of 3 capsules (0.42 g per capsule) three times daily, preferably after meals. (2) The LOPM group: LOPM was performed 3 times per week. (3) The combination (YBT + LOPM) group: Participants received both YBT capsules and LOPM, with the same dosage and manipulation protocol as the YBT group and LOPM group. The procedure of LOPM consisted of three steps: (1) soft tissue assessment to identify the lesion location; (2) tendon-regulation techniques to relax lumbar muscles and soft tissues; and (3) stepwise oblique pull manipulation to mobilize the spine. A quality check was performed after each session. A total of 26 LOPM practitioners (2 per center) delivered the intervention. All practitioners were licensed TCM physicians with at least 5 years of clinical experience. Before the trial, they underwent a standardized training program organized by Wangjing Hospital, China Academy of Chinese Medical Sciences, delivered by physicians with over 20 years of experience in LOPM. A competency assessment was conducted upon completion of training, and only practitioners who met the required standards were permitted to perform the technique.

In addition to their assigned treatments, all participants performed standardized lumbar functional exercises throughout the treatment period. The research team developed standardized exercise instruction manuals, which were distributed to participants upon enrollment. Additionally, study physicians provided in-person training to ensure participants master the exercise techniques. The exercise routine was performed twice daily, consisting of five movements: rotational stretch, swallow dive, supine bridge, knee-to-chest curl, and air cycling. Each session lasted approximately 10 min. Adherence to the prescribed exercises was monitored by direct questioning at each follow-up visit.

All patients received treatment for 2 weeks, and treatment could be discontinued early if symptoms resolve. A comprehensive description of the interventions, including detailed information on the YBT administration, LOPM procedures, and lumbar functional exercise protocols, can be found in Supplementary material 1. If patients required additional medications due to intolerable pain or other reasons, the use of concomitant medications was accurately documented by the researchers.

### Outcomes

Efficacy measures were recorded at baseline and 3 days, 1 week, 2 weeks, 6 weeks, 14 weeks, and 26 weeks after randomization. The measurement instruments used were Oswestry Disability Index (ODI) (21) for the functional disability, VAS for the leg and low back pain, and 12-Item Short Form Health Survey (SF-12) (22) for the quality of life. Blood routine tests and liver and kidney function tests were conducted before randomization and at 2 weeks after randomization. In addition, detailed records of each patient’s gastrointestinal discomfort, skin allergies, and other adverse events were collected at 2 weeks after randomization to assess safety. At the 26-week post-randomization follow-up, a repeat MRI was performed. The maximum area of each disc herniation was measured on sagittal T2-weighted MRI sequences using ImageJ software (National Institutes of Health, Bethesda, MD, USA). All measurements were independently performed by two spine surgeons with extensive experience, and the average of their measurements was taken as the final result. If the difference between the two measurements exceeded 10%, a third senior spine surgeon performed the measurement, and the median of the three measurements was used. The change in the sagittal area of the disc herniation from baseline to 26 weeks was measured, with a reduction of ≥40% defined as the occurrence of disc resorption, the same as the criterion used by Albert et al. (23) The disc resorption rates were calculated. The primary outcome was change in ODI from baseline to 2 weeks. The minimal clinically important difference (MCID) for ODI was set at ≥7 points, consistent with prior studies (24). Secondary outcomes included leg and low back pain VAS, SF-12, adverse events, and disc resorption rates. The data collection methods can be found in Supplementary material 1.

### Sample size

The sample size was calculated using PASS (Power Analysis and Sample Size), version 11 (Number Cruncher Statistical Systems). The calculation was based on detecting a MCID of 7 points on ODI, with a standard deviation of 15.1 points derived from a pre-trial study (24). A statistical power of 90% and a two-sided alpha of 0.05 were assumed. The calculation was based on the primary objective of demonstrating the superiority of the combination therapy over YBT or LOPM alone, using the Dunnett’s test for the two primary comparisons. The initial calculation indicated a requirement of 113 participants per group. After accounting for a 15% dropout rate, the sample size was set at 133 participants per group. To ensure robust multi-center recruitment, the target sample size was further increased to 146 participants per group in the registered protocol. During the prespecified recruitment period, the target of 146 participants per group was not fully achieved. However, the final analyzed sample sizes (146 in the YBT group, 137 in the LOPM group, and 143 in the combination group) exceeded the initial requirement of 133 participants per group calculated *a priori*.

### Statistical analysis

The primary efficacy analysis followed the intention-to-treat (ITT) principle, including all randomly assigned patients. Results from the per-protocol population are presented in Supplementary material 2. All analyses were performed using R software, version 4.4.0 (R Foundation for Statistical Computing). Missing data for the longitudinal outcomes were handled under the assumption of missing at random using multiple imputation. Imputation was performed using the Multivariate Imputation by Chained Equations (MICE) package in R, generating 5 imputed datasets. The imputation model included all longitudinal outcome measures from all-time points and all baseline covariates listed above. Results from the imputed datasets were pooled according to Rubin’s rules. Baseline characteristics were summarized using descriptive statistics. For the continuous outcomes, between-group differences at each specific time point and within-group changes from baseline were evaluated via pairwise comparisons of the estimated marginal means derived from the linear mixed model (LMM), which was fitted using the lme4 package (version 1.1.35). The model included random intercepts for participants. These comparisons were conducted using the emmeans package (version 1.10.0) with Tukey’s method for adjustment of *p* values to control the family-wise error rate. The model included fixed effects for treatment group, time (modeled as a categorical factor), and their interaction. The model was adjusted for the following prespecified baseline covariates: sex, age, body mass index, total duration of lumbar disc disease, duration of the current progressive episode, and degree of herniation at the symptomatic disc level. For categorical outcomes, between-group comparisons were performed using the chi-square test or Fisher’s exact test as appropriate, while the Bonferroni correction was applied to adjust the significance level and control for type I error arising from multiple comparisons. All statistical tests were two-sided, with a *p* value of less than 0.05 considered statistically significant. The analysis period was from August to September 2025. Institute of Basic Research in Clinical Medicine, China Academy of Chinese Medical Sciences performed all statistical analyses. No interim analysis was performed during the trial. The statistical analysis plan was finalized and documented before database lock. All analyses reported herein followed the prespecified statistical analysis plan.

## Results

A total of 426 patients (mean [SD] age, 44.9 [14.1] years; 248 females [58.2%] and 178 males [41.8%]) were included in the analyses, comprising 146 assigned to the YBT group, 137 to the LOPM group, and 143 to the combination group. Baseline demographic and clinical characteristics were well balanced (Table 1). No significant protocol deviations occurred during the trial. For brevity, detailed data on socioeconomic factors (nature of work, income), medical history (comorbidities, lumbar spine trauma), and the full distribution of herniated disc levels are provided in Supplementary Table 1.

**Table 1:** Demographics and baseline characteristics of study participants.

Characteristic	YBT (*n* = 146)	LOPM (*n* = 137)	YBT + LOPM (*n* = 143)
Age, mean (SD), y	43.6 (12.8)	45.8 (14.4)	45.4 (15.1)
Sex, No. (%)			
Female	75 (51.4)	91 (66.4)	82 (57.3)
Male	71 (48.6)	46 (33.6)	61 (42.7)
BMI, mean (SD)	24.4 (4.4)	23.8 (3.3)	24.1 (3.8)
Symptomatic herniated disc level, No. (%)			
L4-L5	84 (57.5)	83 (60.6)	85 (59.4)
L5-S1	37 (25.3)	35 (25.5)	36 (25.2)
Other levels^a^	25 (17.1)	19 (13.9)	22 (15.4)
Degree of herniation at the symptomatic disc level^b^, No. (%)			
Bulge	31 (21.3)	36 (26.3)	36 (25.2)
Protrusion	104 (71.2)	95 (69.3)	102 (71.3)
Extrusion	11 (7.5)	6 (4.4)	5 (3.5)
Duration of disease, mean (SD), d	948.6 (1200.9)	1232.9 (1515.5)	1484.0 (2021.4)
Duration of disease progression, mean (SD), d	40.4 (93.7)	58.9 (168.0)	60.1 (181.9)
ODI^c^, mean (SD)	37.6 (15.3)	38.4 (15.8)	38.8 (16.3)
Leg pain VAS score^d^, mean (SD)	4.5 (1.7)	4.6 (1.5)	4.8 (1.7)
Low back pain VAS score^d^, mean (SD)	5.1 (1.2)	5.0 (1.2)	5.1 (1.1)
SF-12 PCS^e^, mean (SD)	35.6 (7.4)	35.8 (6.5)	36.5 (7.6)
SF-12 MCS^e^, mean (SD)	45.7 (8.5)	45.0 (7.8)	46.4 (7.9)

BMI, body mass index; LOPM, lumbar oblique pull manipulation; MCS, Mental Component Summary; ODI, Oswestry Disability Index; PCS, Physical Component Summary; SD, standard deviation; SF-12, 12-Item Short Form Health Survey; VAS, visual analog scale; YBT, Yaobitong.

a Percentages for “Other levels” (L1-L2, L2-L3, L3-L4) are not shown for brevity; see Supplementary Table 1 for the full distribution.

b Herniation degree was classified on axial and sagittal T2-weighted magnetic resonance imaging (MRI) sequences according to widely accepted morphological criteria: Bulge (circumferential extension >50% of disc circumference); Protrusion (focal herniation with the base wider than the apex); and Extrusion (herniation with the apex wider than the base or the presence of a sequestered fragment).

c ODI assesses the effect of pain on normal daily activity including the ability to and intensity of lifting, care for oneself, walk, sit, sexual function, stand, social life, sleep and travel, ranging from 0 (no disability) to 100 (maximum disability possible).

d Scores for leg pain and low back pain could range from 0 (no pain) to 10 (pain as bad as you can imagine).

e SF-12 assesses the quality of life through physical and mental dimensions, with scores typically ranging from 0 to 100. Higher scores indicate a better quality of life.

### Efficacy

**Figure 3:**
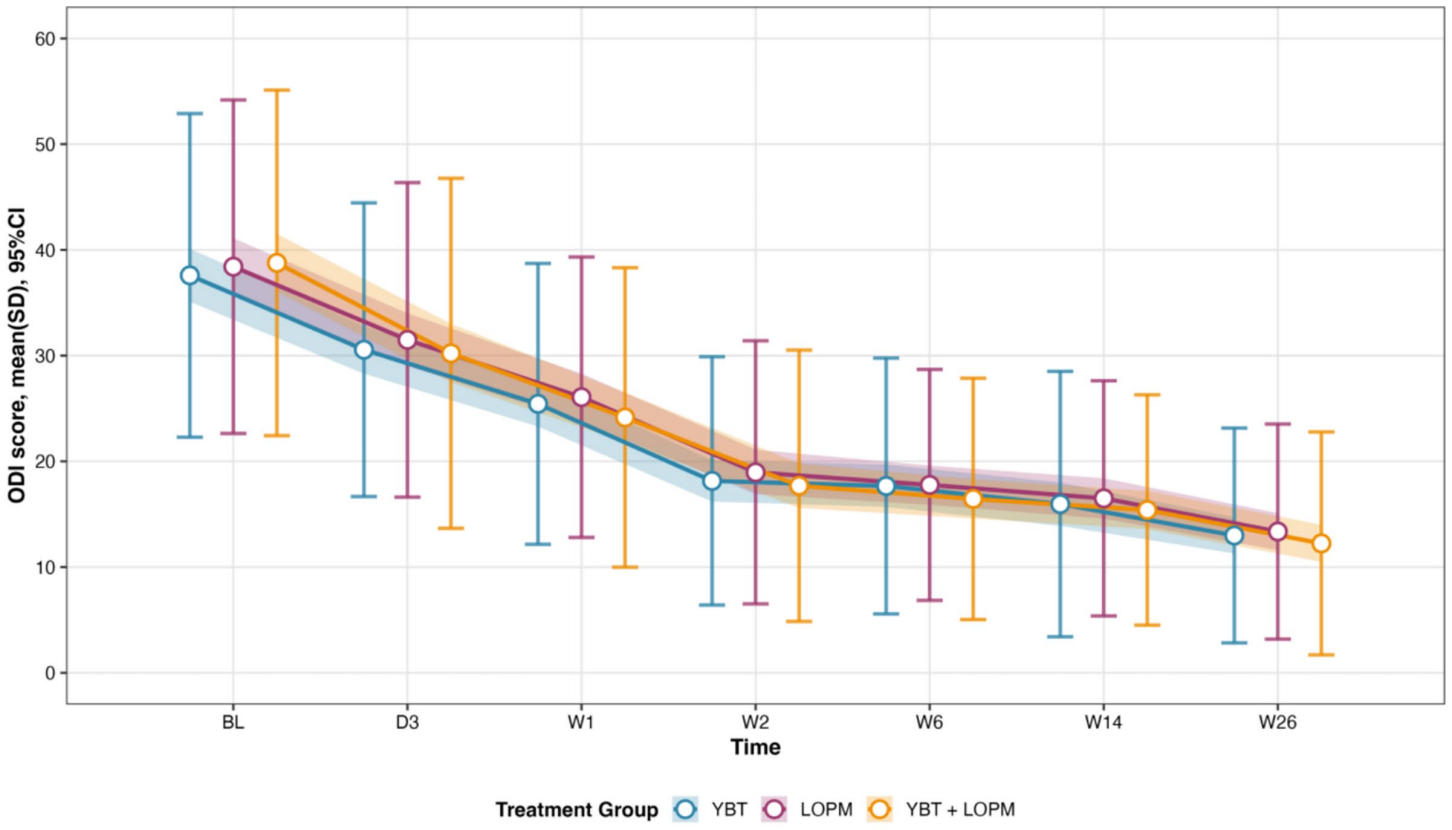
Trajectory of ODI scores over time. BL, baseline; CI, confidence interval; D3, day 3; LOPM, lumbar oblique pull manipulation; ODI, Oswestry Disability Index; SD, standard deviation; W1, week 1; W2, week 2; W6, week 6; W14, week 14; W26, week 26; YBT, Yaobitong.

At 2 weeks, the mean reduction in ODI was −19.4 (95% CI, −21.4 to −17.5) in the YBT group, −19.4 (95% CI, −21.4 to −17.5) in the LOPM group, and −21.1 (95% CI, −23.0 to −19.1) in the combination group. The adjusted mean difference was 0.5 (95% CI, −2.3 to 4.9; *p* = 0.99) for YBT versus combination and 1.3 (95% CI, −2.1 to 5.1, *p* = 0.68) for LOPM versus combination. The 95% CI for both comparisons exclude the prespecified MCID of 7 points, indicating that a clinically meaningful additional benefit of combination therapy is unlikely. No statistically significant differences in ODI reduction were detected between groups at any follow-up time point (all *p* > 0.05) (Table 2; Figure 3; Supplementary Table 2).

**Table 2:** Outcome measures in the intention-to-treat population.

Follow up time	Mean (95%CI)			Difference between groups, mean (95%CI) / *p* value			
	YBT (*n* = 146)	LOPM (*n* = 137)	YBT + LOPM (*n* = 143)	YBT and YBT + LOPM		LOPM and YBT + LOPM	
ODI^a^							
Day 0	37.6 (35.1 to 40.1)	38.4 (35.7 to 41.1)	38.8 (36.1 to 41.5)	−1.2 (−4.0 to 3.3)	0.44	−0.4 (−2.1 to 5.1)	0.97
Day 3	30.6 (28.3 to 32.8)	31.5 (29.0 to 34.0)	30.2 (27.5 to 33.0)	0.3 (−2.3 to 4.9)	0.97	1.3 (−2.0 to 5.2)	0.68
Week 1	25.4 (23.3 to 27.6)	26.1 (23.8 to 28.3)	24.2 (21.8 to 26.5)	1.3 (−1.7 to 5.6)	0.91	1.9 (−2.3 to 4.9)	0.42
Week 2	18.2 (16.2 to 20.1)	19.0 (16.9 to 21.1)	17.7 (15.6 to 19.8)	0.5 (−2.3 to 4.9)	0.99	1.3 (−2.1 to 5.1)	0.68
Week 6	17.7 (15.7 to 19.7)	17.8 (15.9 to 19.6)	16.5 (14.6 to 18.3)	1.2 (−2.3 to 5.0)	0.93	1.3 (−2.8 to 4.4)	0.66
Week 14	16.0 (13.9 to 18.0)	16.5 (14.6 to 18.4)	15.4 (13.6 to 17.2)	0.6 (−2.5 to 4.7)	0.99	1.1 (−2.4 to 4.8)	0.75
Week 26	13.0 (11.3 to 14.7)	13.4 (11.6 to 15.1)	12.2 (10.5 to 14.0)	0.8 (−2.5 to 4.8)	0.99	1.1 (−2.5 to 4.7)	0.74
Leg pain VAS score^b^							
Day 0	4.5 (4.3 to 4.8)	4.6 (4.3 to 4.8)	4.8 (4.5 to 5.1)	−0.3 (−0.7 to 0.2)	0.17	−0.3 (−0.4 to 0.5)	0.37
Day 3	3.9 (3.6 to 4.2)	3.8 (3.5 to 4.0)	4.2 (3.9 to 4.4)	−0.3 (−0.8 to 0.1)	0.17	−0.4 (−0.5 to 0.4)	0.12
Week 1	3.2 (3.0 to 3.5)	3.2 (2.9 to 3.4)	3.4 (3.2 to 3.6)	0.2 (−0.7 to 0.2)	0.42	−0.2 (−0.4 to 0.5)	0.48
Week 2	2.4 (2.1 to 2.7)	2.4 (2.1 to 2.7)	2.5 (2.2 to 2.8)	−0.1 (−0.5 to 0.3)	0.55	−0.1 (−0.3 to 0.5)	0.86
Week 6	2.3 (2.0 to 2.6)	2.4 (2.2 to 2.7)	2.5 (2.3 to 2.8)	−0.2 (−0.5 to 0.4)	0.34	−0.1 (−0.3 to 0.6)	0.92
Week 14	2.1 (1.8 to 2.3)	2.1 (1.9 to 2.4)	2.1 (1.8 to 2.3)	0.0 (−0.4 to 0.5)	0.93	0.0 (−0.3 to 0.6)	0.96
Week 26	1.8 (1.5 to 2.1)	1.8 (1.5 to 2.0)	1.8 (1.6 to 2.0)	0.0 (−0.5 to 0.4)	0.91	−0.1 (−0.4 to 0.5)	0.95
Low back pain VAS score^b^							
Day 0	5.1 (4.9 to 5.3)	5.0 (4.8 to 5.2)	5.1 (4.9 to 5.3)	0.0 (−0.5 to 0.3)	0.93	−0.1 (−0.5 to 0.3)	0.76
Day 3	4.4 (4.2 to 4.6)	4.2 (4.0 to 4.4)	4.3 (4.1 to 4.5)	0.1 (−0.5 to 0.3)	0.96	−0.1 (−0.5 to 0.3)	0.86
Week 1	3.6 (3.3 to 3.8)	3.5 (3.2 to 3.7)	3.5 (3.3 to 3.8)	0.1 (−0.4 to 0.4)	0.99	0.0 (−0.5 to 0.3)	0.97
Week 2	2.6 (2.3 to 2.9)	2.6 (2.3 to 2.9)	2.6 (2.3 to 2.8)	0.1 (−0.4 to 0.4)	0.98	0.1 (−0.4 to 0.4)	0.97
Week 6	2.7 (2.4 to 2.9)	2.6 (2.3 to 2.8)	2.5 (2.3 to 2.8)	0.2 (−0.4 to 0.4)	0.72	0.1 (−0.5 to 0.3)	0.96
Week 14	2.4 (2.2 to 2.7)	2.2 (2.0 to 2.4)	2.2 (1.9 to 2.4)	0.3 (−0.4 to 0.4)	0.36	0.1 (−0.6 to 0.2)	0.98
Week 26	2.0 (1.8 to 2.2)	1.9 (1.7 to 2.2)	1.9 (1.6 to 2.1)	0.1 (−0.3 to 0.5)	0.73	0.1 (−0.5 to 0.4)	0.88
SF-12 PCS^c^							
Day 0	35.7 (34.4 to 36.9)	35.8 (34.7 to 36.9)	36.5 (35.2 to 37.8)	−0.9 (−2.7 to 1.4)	0.55	−0.7 (−1.8 to 2.3)	0.72
Day 3	36.8 (35.6 to 38.0)	37.6 (36.4 to 38.7)	38.0 (36.7 to 39.3)	−1.2 (−2.5 to 1.6)	0.31	−0.4 (−1.2 to 2.9)	0.88
Week 1	38.4 (37.2 to 39.6)	39.2 (38.1 to 40.3)	40.0 (38.8 to 41.2)	−1.6 (−2.9 to 1.2)	0.13	−0.8 (−1.2 to 2.9)	0.61
Week 2	40.9 (39.6 to 42.2)	41.7 (40.6 to 42.8)	41.9 (40.6 to 43.2)	−1.0 (−2.2 to 1.9)	0.44	−0.2 (−1.2 to 2.9)	0.98
Week 6	43.1 (41.9 to 44.3)	43.4 (42.3 to 44.6)	43.0 (41.7 to 44.2)	0.2 (−1.6 to 2.5)	0.99	0.5 (−1.7 to 2.4)	0.84
Week 14	44.1 (42.8 to 45.3)	44.6 (43.3 to 45.8)	44.3 (43.0 to 45.5)	−0.2 (−1.7 to 2.4)	0.95	0.3 (−1.5 to 2.6)	0.93
Week 26	45.0 (43.7 to 46.2)	45.2 (44.0 to 46.4)	44.7 (43.5 to 45.9)	0.3 (−1.5 to 2.6)	0.96	0.5 (−1.7 to 2.4)	0.80
SF-12 MCS^c^							
Day 0	45.7 (44.3 to 47.1)	44.96 (43.6 to 46.3)	46.4 (45.1 to 47.7)	−0.7 (−3.6 to 0.7)	0.74	−1.4 (−3.0 to 1.3)	0.24
Day 3	46.1 (44.8 to 47.4)	46.1 (44.8 to 47.4)	47.3 (46.0 to 48.6)	−1.2 (−3.4 to 0.9)	0.38	−1.2 (−2.2 to 2.1)	0.34
Week 1	47.9 (46.6 to 49.1)	47.6 (46.3 to 48.9)	47.8 (46.5 to 49.0)	0.1 (−2.4 to 1.9)	0.99	−0.2 (−2.6 to 1.8)	0.97
Week 2	48.7 (47.4 to 50.0)	47.9 (46.6 to 49.1)	49.2 (47.9 to 50.4)	−0.4 (−3.5 to 0.8)	0.87	−1.3 (−3.1 to 1.2)	0.28
Week 6	50.0 (48.9 to 51.2)	48.8 (47.5 to 50.1)	49.5 (48.3 to 50.7)	0.5 (−2.9 to 1.4)	0.84	−0.7 (−3.5 to 0.8)	0.66
Week 14	50.3 (49.2 to 51.5)	49.4 (48.1 to 50.7)	50.4 (49.2 to 51.5)	0.0 (−3.2 to 1.1)	0.99	−1.0 (−3.2 to 1.1)	0.49
Week 26	51.2 (50.0 to 52.4)	50.1 (48.8 to 51.4)	51.0 (49.8 to 52.2)	0.2 (−3.1 to 1.2)	0.98	−0.9 (−3.2 to 1.1)	0.54

CI, confidence interval; LOPM, lumbar oblique pull manipulation; MCS, Mental Component Summary; ODI, Oswestry Disability Index; PCS, Physical Component Summary; SF-12, 12-Item Short Form Health Survey; VAS, visual analog scale; YBT, Yaobitong.

a ODI assesses the effect of pain on normal daily activity including the ability to and intensity of lifting, care for oneself, walk, sit, sexual function, stand, social life, sleep and travel, ranging from 0 (no disability) to 100 (maximum disability possible).

b Scores for leg pain and low back pain could range from 0 (no pain) to 10 (pain as bad as you can imagine).

c SF-12 assesses the quality of life through physical and mental dimensions, with scores typically ranging from 0 to 100. Higher scores indicate a better quality of life.

No statistically significant differences in any of the secondary outcomes were detected between groups at any follow-up time point (all *p* > 0.05) (Table 2; Supplementary Table 2; Supplementary Figures 1–4).

At 2 weeks, the proportion of patients achieving the MCID was 84.2% (95% CI, 77.5 to 89.3%) in the YBT group, 81.8% (95% CI, 74.5 to 87.3%) in the LOPM group, and 87.4% (95% CI, 81.0 to 91.9%) in the combination group. No statistically significant between-group differences were observed (YBT vs. combination: *p* = 0.44; LOPM vs. combination: *p* = 0.19) (Table 3).

**Table 3:** Proportion of patients achieving the minimal clinically important difference (≥7-point Oswestry Disability Index reduction) at week 2 in the intention-to-treat population.

No. (%) (95% CI)			Difference between groups, *p* value	
YBT (*n* = 146)	LOPM (*n* = 137)	YBT + LOPM (*n* = 143)	YBT and YBT + LOPM	LOPM and YBT + LOPM
123 (84.2) (77.5 to 89.3)	112 (81.8) (74.5 to 87.3)	125 (87.4) (81.0 to 91.9)	0.44	0.19

CI, confidence interval; LOPM, lumbar oblique pull manipulation; YBT, Yaobitong.

The results of the per-protocol analysis were generally consistent with those of the intention-to-treat population (Supplementary Tables 3, 4).

### Adverse events

In the combination group, 2 (1.4%) adverse events occurred: one case of skin allergy (onset on day 4 and resolved in 3 days) and one case of abdominal pain with diarrhea (onset on day 2 and resolved in 2 days). Both adverse events were mild in severity, non-serious, and considered possibly related to treatment. Neither event required medical intervention (Supplementary Table 5). No adverse events were reported in the YBT or LOPM groups. Blood routine tests and liver/kidney function tests performed at baseline and week 2 showed no clinically relevant abnormalities in any group.

### Intervention adherence and concomitant interventions

Specific adherence rates for YBT capsules, LOPM sessions, and lumbar functional exercises were not systematically recorded. However, at each follow-up visit, researchers inquired about adherence and provided encouragement to promote compliance. No participants discontinued treatment prematurely. No rescue or concomitant medications were used in any group.

### Disc resorption rates

At 26 weeks, MRI scans were successfully obtained from 96 (65.8%) participants in the YBT group, 90 (65.7%) in the LOPM group, and 96 (67.1%) in the combination group. The reasons for missing MRI data are summarized in Supplementary Table 6. The proportion of patients achieving disc resorption was 12.5% (95% CI, 7.3 to 20.6%) in the YBT group, 7.8% (95% CI, 3.8 to 15.2%) in the LOPM group, and 10.4% (95% CI, 5.8 to 18.1%) in the combination group. No significant between-group differences were observed in disc resorption rates (YBT vs. combination: *p* = 0.65; LOPM vs. combination: *p* = 0.53) (Table 4).

**Table 4:** Proportion of patients achieving the disc resorption at week 26.

No. (%) (95% CI)			Difference between groups, *p* value	
YBT (*n* = 96)	LOPM (*n* = 90)	YBT + LOPM (*n* = 96)	YBT and YBT + LOPM	LOPM and YBT + LOPM
12 (12.5) (7.3 to 20.6)	7 (7.8) (3.8 to 15.2)	10 (10.4) (5.8 to 18.1)	0.65	0.53

CI, confidence interval; LOPM, lumbar oblique pull manipulation; YBT, Yaobitong.

## Discussion

This multicenter, three-arm, parallel-group RCT demonstrated that, over a 2-week intervention period, combination therapy with YBT and LOPM did not confer statistical superiority over either monotherapy across all efficacy outcomes assessed in LDHR patients with moderate pain.

YBT is primarily indicated for activating blood circulation, resolving stasis, and promoting qi movement to alleviate pain. Pharmacological studies have demonstrated its multi-target anti-inflammatory and analgesic effects (25, 26). Clinically, YBT has been shown to produce significant improvements in ODI and VAS scores among patients with LDHR (11). Current evidence supports the role of YBT in managing LDHR (8). LOPM restores lumbar function and enhances spinal stability through a series of biomechanical effects, including relaxing lumbar muscles, mobilizing spinal joints, improving soft tissue tension, reducing intradiscal pressure, increasing the volume of the nerve root canal, and releasing adhesions within the facet joints, thereby alleviating nerve root compression. From the perspective of TCM theory, LOPM addresses the manifestations by regulating tendons, restoring bone structure, and unblocking meridians to directly relieve local qi and blood stagnation. Clinical studies (27) and systematic reviews (12, 28) have confirmed the significant clinical benefits and favorable safety profile of this technique in relieving pain and improving function among patients with LDHR.

However, our study did not confirm the superiority of combination therapy over either monotherapy. The CI exclude a clinically relevant advantage, thereby strengthening the conclusion that combination therapy is unlikely to provide meaningful incremental benefit. A potential explanation for this finding may relate to the highly effective response observed with monotherapy itself. Within the 2-week treatment period, ODI scores improved by 19.4 points in the YBT group and 19.0 points in the LOPM group, both far exceeding the predefined MCID of 7 points. Against this backdrop of highly effective monotherapy, although the combination therapy showed numerically greater improvement (21.1 points), the incremental effect size (approximately 2 points) was substantially smaller than the anticipated between-group difference (7 points). This resulted in the modest advantage of the combination group not being statistically demonstrated. In addition, YBT and LOPM may share overlapping therapeutic mechanisms, which could limit the potential for additive or synergistic effects when combined. The relatively short intervention duration (2 weeks) may also have been insufficient to allow any true additive effect to become clinically detectable. Finally, the natural course of LDHR includes a substantial potential for spontaneous improvement, and the standardized lumbar functional exercises received by all participants may have contributed to the observed improvements, further reducing the detectable added value of combination therapy.

Although numerous previous studies have demonstrated that combining certain interventions can offer therapeutic advantages over monotherapy in spinal disorders (15, 29), our study found no such superiority for the combination of YBT and LOPM in LDHR patients with moderate pain. The absence of synergistic benefit observed in our study is not an isolated finding. A recent RCT evaluating the addition of an herbal compress to traditional Thai massage for elderly patients with chronic low back pain similarly reported no statistically significant differences between the combination and massage-only groups across all primary and secondary outcomes, despite both groups demonstrating substantial within-group improvements (30). The findings provide an important clinical insight: the synergistic effect of combination therapy is not automatic; rather, its benefits may vary depending on the disease type, specific interventions, and patient population. Therefore, when making treatment decisions, clinicians should adopt a prudent and individualized approach. In the absence of superior efficacy of combination therapy, monotherapy with either YBT or LOPM may be reasonably chosen based on cost, availability, patient preference, safety considerations, and treatment burden. For example, YBT may be preferred in settings where access to trained LOPM practitioners is limited, while LOPM may be preferred by patients seeking non-pharmacological options or those concerned about potential drug-related issues.

Previous studies in this area have frequently suffered from methodological limitations, leading to a low overall quality of evidence (8). By contrast, our trial was designed through an iterative process involving multiple rounds of expert consultation (Supplementary material 1), strictly followed international research standards, and utilized validated, widely accepted outcome instruments. Additionally, MRI assessments were incorporated to evaluate structural changes in the intervertebral disc, providing an objective imaging-based component to the outcome evaluation. Randomization and all statistical analyses were carried out independently by an external third-party institution. The trial was conducted across 8 provinces and 3 municipal districts in China, thereby strengthening the external validity of its findings.

Several limitations of this study should be acknowledged. (1) A true control group was not included for ethical reasons. Given that LDHR exhibits a certain potential for spontaneous improvement, the absence of such a control makes it difficult to precisely isolate the specific effects of the interventions from natural recovery processes. Additionally, all participants received standardized lumbar functional exercises, and the absence of an exercise-only control group means that the contribution of the exercise program cannot be separated from the effects of YBT or LOPM. (2) The core outcomes (ODI, VAS, and SF-12) were assessed using patient-reported instruments, as objective measurement tools for these fundamental components of LDHR are currently lacking. Similarly, adherence to lumbar functional exercises was monitored by direct questioning, which may be subject to recall bias. (3) Complete blinding of participants and therapists was not achievable. Due to the hands-on nature of manual therapy, masking the manipulation procedure was inherently impossible. Although YBT is an oral agent for which blinding could, in principle, be accomplished through a placebo-controlled design, inactive placebo capsules were not employed. This limitation may have introduced bias in patient-reported outcomes, expectation effects and performance bias can meaningfully influence subjective outcomes when participants know their treatment assignment. Blinding of outcome assessors and statisticians, while valuable, does not fully address this issue. Therefore, this study should be viewed as a pragmatic comparative-effectiveness trial rather than a placebo-controlled efficacy trial. (4) Despite all LOPM practitioners undergoing standardized training and rigorous evaluation prior to the study, some degree of variation in technique execution across centers and individual therapists cannot be entirely excluded. Such variability could have influenced the consistency of treatment effects. We did not formally test for variability in outcomes by center or individual practitioner. (5) The 26-week MRI assessment had incomplete follow-up (approximately 34% missing). We did not compare baseline characteristics between participants with and without MRI data. Disc resorption findings should be interpreted as exploratory. (6) Although the final sample size exceeded the initial calculation requirement of 133 participants per group, the registered target of 146 per group was not achieved. This shortfall may have reduced the precision of the estimates. (7) Subgroup analyses based on baseline pain severity, herniation type, disease duration, age, or sex were not performed. Future studies may explore these potential effect modifiers. (8) The intervention duration was only 2 weeks, which may be insufficient to detect potential synergistic effects that could require longer exposure to manifest.

## Conclusion

This multicenter RCT demonstrated that, over a 2-week intervention period, combination therapy with YBT and LOPM was not superior to either monotherapy in improving functional disability, pain, or quality of life among LDHR patients with moderate pain.

## Data Availability

The original contributions presented in the study are included in the article/Supplementary material, further inquiries can be directed to the corresponding authors.

## Glossary

BMI: Body mass index
CI: Confidence interval
COA: Chinese Orthopaedic Association
CONSORT: Consolidated Standards of Reporting Trials
CRF: Case report form
CT: Computed tomography
ICF: Informed consent form
ITT: Intention-to-treat
LDHR: Lumbar disc herniation with radiculopathy
LMM: Linear mixed model;
LOPM: Lumbar oblique pull manipulation
MCID: Minimal clinically important difference
MCS: Mental Component Summary
MICE: Multivariate Imputation by Chained Equations
MRI: Magnetic resonance imaging
NASS: North American Spine Society
NMPA: National Medical Products Administration
ODI: Oswestry Disability Index
PASS: Power Analysis and Sample Size
PCS: Physical Component Summary
RCT: Randomized controlled trial
SD: Standard deviation
SF-12: 12-Item Short Form Health Survey
TCM: Traditional Chinese Medicine
VAS: Visual analog scale
YBT: Yaobitong
