# Supplementary material for: Combination of Yaobitong capsules and lumbar oblique pull manipulation for moderate pain in lumbar disc herniation with radiculopathy: a multicenter, randomized, three-arm, parallel-group controlled trial
Source: Front Neurol. 2026 Jun 26;17:1853703. doi: 10.3389/fneur.2026.1853703 (PMC13349874; doi:10.3389/fneur.2026.1853703)
Supplement: Supplementary file 1 [file Supplementary_file_1.pdf]

Clinical Evaluation of TCM Regimens for Lumbar Disc Herniation with Radiculopathy

(Study Protocol)

Date of protocol: June 30, 2022

Principal investigator: Zhu Li-Guo, MD., PhD., Professor in Wangjing Hospital, China Academy of Chinese Medical Sciences

This protocol is an intellectual property of the Wangjing Hospital, China Academy of Chinese Medical Sciences. All relevant study information is confidential and can be neither copied nor disclosed to anyone or any institution except the study personnel, ethics committee, or relevant regulatory departments of the study institution involved in this clinical study.

Table of contents

Study site and responsible person....4
Executive committee member....4
Clinical endpoint committee members....5
Data and safety monitoring board....5
Third-party data statistical unit....5
Sponsor and contact information....5
Principal study site and contact information....5
Abbreviation....6
Summary....7
Study protocol....9
1. Background....9
2. Objective....9
3. Study design....9
3.1. Randomization and Masking....10
3.2. Sample size....10
4. Study population....10
4.1. Diagnostic criteria....10
4.2. Inclusion criteria....11
4.3. Exclusion criteria....11
4.4. Drop-out criteria....11
4.5. Discontinuation criteria....12
4.6. Termination criteria....12
5. Treatment....12
5.1. Treatment protocol....13
5.11. Description of treatment methods....13
5.2. Concomitant medication protocol....20
5.3. Drug dispensing and storage protocol....20
5.4. Investigational drug management....21
5.5. Quality control (QC) and monitoring of investigational drugs....22
5.5.1. Drug QC....22
5.5.2. Expiry date management of investigational drugs....22
5.6. Adverse drug reactions (ADRs) and reporting....22
5.7. Emergency drug management protocol....23
6. Observation endpoints and clinical outcomes....23

6.1. Clinical observation endpoints....23
6.2. Safety assessment....27
6.3. Disc resorption assessment....27
7. AEs observation and analysis....28
7.1. Criteria for AE assessment....28
7.2. Analysis of causality and severity....28
7.3. Documentation of common AE....29
7.4. Management of AE....29
7.5. Analysis of AE....30
7.6. Abnormal laboratory findings....30
8. QC and quality assurance....30
8.1. Analysis of study influencing factors....30
8.2. Infrastructure for QC....31
8.3. Study training and monitoring protocols....32
8.4. Strategies to improve participant compliance....32
8.5. QC and quality assurance system....33
9. Data administration....34
9.1. Source data documentation....35
9.2. Data recording and reporting standards....35
9.3. Source document archiving standards....35
9.4. Source document access regulations....35
9.5. Data verification protocol....36
9.6. CRF completion guidelines....36
9.7. Electronic data reporting protocol....37
9.8. Database review and locking procedures....38
10. Statistical analysis....38
10.1 Basis for protocol development....38
10.2 Analysis populations....39
10.3 Safety analysis population....39
10.4 ITT....39
10.5 PP....39
10.6 Statistical method....39
11. Ethical issues....40
11.1. Ethic review....40
11.2. Ethical review system....40

11.3. Benefits and risks assessment....41
11.3.1 Potential benefits....41
11.3.2 Anticipated risks....41
11.3.3 Risk mitigation strategies....41
11.4. Informed consent process....41
11.4.1 Pre-enrollment requirements....42
11.4.2 Special circumstances....42
11.5. Participant recruitment procedures....42
11.5.1 Recruitment methods....42
11.5.2 Eligibility verification....42
11.5.3 Ethical compliance....42
11.6. Participant rights and protections....42
11.6.1 Voluntary participation & withdrawal rights....43
11.6.2 Medical record handling....43
11.6.3 Privacy safeguards....43
11.7. EC continuing review....43
11.7.1 Scope and frequency of review....43
11.7.2 Review procedures....44
11.7.3 Regulatory compliance....44
11.8. EC inspection procedures....44
11.8.1 Roles and responsibilities....44
11.8.2 Inspection readiness....45
12. Storage and archiving of research materials....45
13. Clinical summary....45
14. Research participating institutions and task allocation....46
14.1 Allocation of research tasks....46
14.2 Allocation of enrolled patients....46
15. References....48

Study site and responsible person

**Principle study site**	Wangjing Hospital, China Academy of Chinese Medical Sciences
**Principle investigator**	Liguo Zhu
**General coordinator of the project**	Jie Yu, Minghui Zhuang
**Participating institutions**	Affiliated Hospital of Changchun University of Chinese Medicine
	Affiliated Hospital of Shaanxi University of Chinese Medicine
	Affiliated Hospital of Shandong First Medical University
	Dongzhimen Hospital, Beijing University of Chinese Medicine
	First Teaching Hospital of Tianjin University of Traditional Chinese Medicine
	Gansu Provincial Hospital of Traditional Chinese Medicine
	Guangdong Provincial Hospital of Chinese Medicine
	Liaoning University of Traditional Chinese Medicine
	Luoyang Orthopedic-Traumatological Hospital of Henan Province
	Nanfang Hospital, Southern Medical University
	Suzhou Hospital of Traditional Chinese Medicine
	The First Hospital of Hunan University of Chinese Medicine
	Jiangsu Kanion Pharmaceutical Co., Ltd

Executive committee member

**Committee member**	**Site**
Liguo Zhu	Wangjing Hospital, China Academy of Chinese Medical Sciences
Shaojun Li	Affiliated Hospital of Changchun University of Chinese Medicine
Puwei Yuan	Affiliated Hospital of Shaanxi University of Chinese Medicine
Bin Shi	Affiliated Hospital of Shandong First Medical University
Yusong Jia	Dongzhimen Hospital, Beijing University of Chinese Medicine
Ping Wang	First Teaching Hospital of Tianjin University of Traditional Chinese Medicine
Jirong Zhao	Gansu Provincial Hospital of Traditional Chinese Medicine
Bolai Chen	Guangdong Provincial Hospital of Chinese Medicine
Xuefeng Guan	Liaoning University of Traditional Chinese Medicine
Jiayi Guo	Luoyang Orthopedic-Traumatological Hospital of Henan Province
Yikai Li	Nanfang Hospital, Southern Medical University
Hong Jiang	Suzhou Hospital of Traditional Chinese Medicine
Shaofeng Yang	The First Hospital of Hunan University of Chinese Medicine
Yanming Xie	Institute of Basic Research in Clinical Medicine, China Academy of Chinese Medical Sciences

Clinical endpoint committee members

Committee member	Site
Xu Wei	Wangjing Hospital, China Academy of Chinese Medical Sciences
Wei Cao	Wangjing Hospital, China Academy of Chinese Medical Sciences
Shangquan Wang	Wangjing Hospital, China Academy of Chinese Medical Sciences
Changlong Shi	Wangjing Hospital, China Academy of Chinese Medical Sciences
Jia Ma	Wangjing Hospital, China Academy of Chinese Medical Sciences

Data and safety monitoring board

Committee member	Site
Jinghua Gao	Wangjing Hospital, China Academy of Chinese Medical Sciences
Yuwei Li	Suzhou Hospital of Traditional Chinese Medicine
Zhenhua Li	Affiliated Hospital of Changchun University of Chinese Medicine
Zhaolan Liu	Beijing University of Chinese Medicine
Wei Chen	Beijing University of Chinese Medicine

Third-party data statistical unit

Data statistical unit	Responsible person	Email
Institute of Basic Research in Clinical Medicine, China Academy of Chinese Medical Sciences	Yanming Xie	ktzu2018@163.com

Sponsor and contact information

Sponsor	Contact person	Email
Chinese National Center for Biotechnology Development, Ministry of Science and Technology of the People's Republic of China	Zihan Fang	fangzh@cncbd.org.cn

Principal study site and contact information

Site	Contact person	Email
Wangjing Hospital, China Academy of Chinese Medical Sciences	Jie Yu	18800122947@163.com
	Minghui Zhuang	a20110134@sina.com

Abbreviation

Abbreviation	Full term
ADR	Adverse drug reaction
AE	Adverse event
CRF	Case report form
EC	Ethics committee
EDC	Electronic data capture
GCP	Good clinical practice
IRB	Institutional review board
ITT	Intention-to-treat
LDHR	Lumbar disc herniation with radiculopathy
LMM	Linear mixed model
LOPM	Lumbar oblique pull manipulation
MCID	Minimal clinically important difference
NSAIDs	Non-steroidal anti-inflammatory drugs
ODI	Oswestry Disability Index
PI	Principal investigator
PP	Per-protocol
QC	Quality control
RCT	Randomized controlled trial
SAE	Serious adverse event
SF-12	12-Item Short Form Health Survey
SR	Sustained release
TCM	Traditional Chinese medicine
VAS	Visual analog scale
YBT	Yaobitong

Summary

**Title**	Clinical Evaluation of TCM Regimens for Lumbar Disc Herniation with Radiculopathy.
**Objective**	To evaluate the therapeutic effect in combination of Yaobitong (YBT) and lumbar oblique pull manipulation (LOPM) for Lumbar disc herniation with radiculopathy (LDHR) patients with moderate pain.
**Study design**	Multicenter, randomized, three-arm, parallel-group controlled trial.
**Inclusion and exclusion criteria**	**Inclusion Criteria:**<br>1.Confirmed diagnosis of LDHR.<br>2.Aged 18–65 years (inclusive).<br>3.Moderate low back or leg pain intensity assessed by visual analog scale (VAS): $\geqslant$ 4 and $<$ 7.<br>**Exclusion Criteria:**<br>1.History of spinal surgery.<br>2.Spinal compression fractures; lumbar spondylolisthesis grade II or higher; lumbar spondylolysis; lumbar spinal stenosis.<br>3.Spinal tumors; spinal tuberculosis; severe osteoporosis (T-score $\leqslant$ -3.0 or associated osteoporotic fractures); diabetes with peripheral neuropathy.<br>4.Known allergy or hypersensitivity to non-steroidal anti-inflammatory drugs (NSAIDs), Chinese herbal medicines, or other related medications.<br>5.History of gastrointestinal ulcer or bleeding.<br>6.Recent coronary artery bypass graft surgery; current use of dual antiplatelet therapy; presence of coagulation disorders or high risk of bleeding.<br>7.Severe skin diseases or skin lesions in the lumbar region.<br>8.Pregnancy or breastfeeding.<br>9.Severe heart failure, stroke, or other major cardiovascular or cerebrovascular diseases; severe hepatic or renal dysfunction.<br>10.Cauda equina syndrome; lower limb muscle strength of grade 3 or less (on a 0-5 scale); or persistent loss of motor/sensory function with clear indications for surgical intervention.<br>11.Inability to provide informed consent or comply with study procedures (e.g., significant visual, hearing, or speech impairment; intellectual disability; or severe mental disorder).
**Endpoints**	Efficacy measures will be recorded at baseline and 3 days, 1 week, 2 weeks, 6 weeks, 14 weeks, and 26 weeks after randomization. The measurement instruments will be Oswestry Disability Index (ODI) for the functional disability, VAS for the leg and low back pain, and 12-Item Short Form Health Survey (SF-12) for the quality of life. Blood routine tests and liver and kidney function tests will be conducted before randomization and at 2 weeks after randomization. In addition, detailed records of each patient's gastrointestinal discomfort, skin allergies, and other adverse events will be collected at 2 weeks after randomization to assess safety. At the 26-week post-randomization follow-up, a repeat MRI will be performed. The change in the sagittal area of the disc herniation from baseline to 26 weeks will be measured, with a reduction of ≥40% defined as the occurrence of disc resorption. The disc resorption rates will be calculated. The primary outcome will be change in ODI from baseline to week 2. The minimal clinically important difference (MCID) for ODI will be set at ≥7 points, consistent with prior studies. Secondary outcomes will include leg and low back pain VAS, SF-12, adverse events, and disc resorption rates.

**Safety indicators**	Blood routine tests and liver and kidney function tests will be conducted at baseline (before randomization) and at 2 weeks after randomization. Additionally, detailed records of gastrointestinal discomfort, skin allergies, and other adverse events will be collected at the 2-week follow-up to assess safety.
**Sample size**	A total of 438 LDHR patients with moderate pain will be included.
**Intervention**	Participants were randomly assigned in a 1:1:1 ratio to one of three treatment groups: (1) The YBT group: YBT capsules were administered orally. (2) The LOPM group: LOPM was performed. (3) The YBT + LOPM group: Participants received both YBT and LOPM.
**Study duration**	2-week treatment and 24-week follow-up.
**Statistical unit**	Institute of Basic Research in Clinical Medicine, China Academy of Chinese Medical Sciences.
**Expected progression**	The trial will commence patient enrollment in January 2023, with complete enrollment and follow-up anticipated by December 2024. Data compilation and statistical analysis are projected to be completed by April 2025.

Study protocol

1. Background

Globally, low back pain represents a paramount burden to public health, ranking as the leading cause of productivity loss and the foremost contributor to years lived with disability in high- and middle-income nations.[1,2] Lumbar disc herniation with radiculopathy (LDHR) is one of the most common causes of low back pain. It is roughly estimated that around 1%-3% of the population suffer from LDHR annually.[3] Alarmingly, the trend is worsening due to an aging population and evolving lifestyles, with LDHR now affecting younger individuals at an increasing rate. This escalating prevalence underscores the critical need for effective prevention and management strategies to alleviate the substantial burden on patients and healthcare systems.

Treatment options for LDHR include both surgical and non-surgical approaches, with the choice primarily dependent on symptom severity. In certain clinical scenarios, such as cauda equina syndrome, immediate surgical intervention is mandatory. While surgery is effective, it is associated with substantial costs and potential risks. Non-surgical management is considered the first-line option for the majority, particularly during the initial 6-week treatment period, especially for those presenting with moderate pain.[4,5]

For LDHR, Traditional Chinese Medicine (TCM) modalities, notably Chinese patent medicine and manual therapy have shown potential in improving patients' quality of life, alleviating discomfort, and restoring motor function, with a favorable safety profile. Yaobitong (YBT) capsule, a Chinese patent medicine approved by the National Medical Products Administration (NMPA) for treating LDHR, is widely used in orthopedic clinical practice in China. Similarly, lumbar oblique pull manipulation (LOPM) is a characteristic manual therapy technique extensively employed in Chinese orthopedic and rehabilitation settings.

In recent years, a growing body of research has suggested that combining internal and external therapies may produce synergistic effects in the management of musculoskeletal disorders. However, despite the widespread combined use of YBT and LOPM for LDHR in Chinese clinical practice, no randomized controlled trial systematically evaluated the efficacy of this combination therapy.

Therefore, focusing on LDHR patients with moderate pain, we will conduct a multicenter, randomized, three-arm parallel-group controlled trial to evaluate the efficacy and safety of combination of YBT and LOPM.

2. Objective

To evaluate the therapeutic effect in combination of YBT and LOPM for LDHR patients with moderate pain.

3. Study design

This is a multicenter, randomized, three-arm, parallel-group controlled trial that will be conducted at 13 tertiary hospitals in China.

3.1. Randomization and Masking

An independent third party, the Institute of Basic Research in Clinical Medicine at the China Academy of Chinese Medical Sciences, will generate the randomization sequence and oversee the masking procedures. A stratified block randomization method will be employed, using study center as stratification factors. Random sequences with a block size of 6 will be generated using SAS, version 9.4 (SAS Institute). Allocation concealment will be ensured through a password-protected, central online randomization system. Investigators will be able to access the treatment assignment only after a patient has completed eligibility confirmation and has provided written informed consent.

3.2. Sample size

The sample size was calculated using PASS (Power Analysis and Sample Size), version 11 (Number Cruncher Statistical Systems). The calculation was based on detecting a MCID of 7 points on Oswestry Disability Index (ODI)[6], with a standard deviation of 15.1 points derived from a pre-trial study[7]. A statistical power of 90% and a two-sided alpha of 0.05 were assumed. The calculation was based on the primary objective of demonstrating the superiority of the combination therapy over YBT or LOPM alone, using the Dunnett's test for the two primary comparisons. The initial calculation indicated a requirement of 113 participants per group. After accounting for a 15% dropout rate, the sample size was set at 133 participants per group. To ensure robust multi-center recruitment, the target sample size was further increased to 146 participants per group.

4. Study population

To be enrolled, patients will need to meet all inclusion criteria and none of the exclusion criteria.

4.1. Diagnostic criteria

The diagnosis of LDHR is established based on the clinical guidelines from the North American Spine Society (NASS)[8] and the Chinese Orthopaedic Association (COA).[9]

(1) Medical history: Presence or absence of definitive lumbar injury history.

(2) Symptoms and signs: Low back pain radiating to the buttock and lower extremity, exacerbated by increased abdominal pressure (e.g., coughing, sneezing); Tenderness at the affected vertebral level with radiation to the lower extremity and restricted lumbar mobility; Hypersensitivity or numbness in the affected nerve dermatome; chronic cases may present with muscle atrophy, diminished tendon reflexes, and/or weakened extensor hallucis longus strength.

(3) Physical examination: Motor or sensory deficits in the affected nerve distribution Positive femoral nerve stretch test Positive straight leg raise test (SLRT), positive SLRT reinforcement test, or positive contralateral SLRT.

(4) Imaging findings: MRI-confirmed LDHR at levels consistent with clinical manifestations.

4.2. Inclusion criteria

(1) Confirmed diagnosis of LDHR.

(2) Aged 18-65 years (inclusive).

(3) Moderate low back or leg pain intensity assessed by visual analog scale (VAS)[10]: ⩾ 4 and < 7.

4.3. Exclusion criteria

(1) History of spinal surgery.

(2) Spinal compression fractures; lumbar spondylolisthesis grade II or higher; lumbar spondylolysis; lumbar spinal stenosis.

(3) Spinal tumors; spinal tuberculosis; severe osteoporosis (T-score ≤ -3.0 or associated osteoporotic fractures); diabetes with peripheral neuropathy.

(4) Allergies to non-steroidal drugs, Chinese medicine, or other related medications.

(5) History of gastrointestinal ulcers/bleeding.

(6) Patients undergoing coronary artery bypass surgery; those on dual-antiplatelet therapy or with coagulation disorders, or those with a high risk of bleeding.

(7) Severe skin diseases or skin lesions in the lumbar region.

(8) Pregnant or breastfeeding women.

(9) Severe heart failure, stroke, or other severe cardiovascular and cerebrovascular diseases; severe liver or kidney dysfunction.

(10) Cauda equina injury, lower limb muscle strength ≤3, or persistent loss of lower limb motor or sensory function with indications for surgery.

(11) Special populations who are unsuitable for clinical trials (e.g., blind, deaf, mute, or those with intellectual or mental disorders).

4.4. Drop-out criteria

Participants who discontinue the study for any reason will be considered dropouts. Voluntary withdrawal will be permitted due to personal reasons, health concerns, or loss of confidence in the study protocol. Investigators will retain the right to withdraw participants for protocol violations, poor compliance, or deteriorating health status that makes continued participation inadvisable. For all dropouts, investigators will perform final safety and efficacy assessments whenever possible and document withdrawal reasons. All data from withdrawn participants will be included in the final intention-to-treat (ITT) analysis to maintain data integrity.

4.5. Discontinuation criteria

Participants will be discontinued from the study under any of the following circumstances:

(1) Adverse event (AE): Discontinuation will be required if a participant experiences:

1) A severe allergic reaction (e.g., anaphylaxis, angioedema) deemed related to the study intervention.

2) Any other drug-related AE that, in the investigator's judgment, poses a significant health risk or precludes further participation.

(2) Pregnancy: Confirmation of pregnancy during the study period will mandate immediate discontinuation.

(3) Participant Decision: Participants will be able to voluntarily withdraw from the study at any time, for any reason, without prejudice to their future medical care.

(4) Investigator-Initiated Discontinuation: The investigator will be able to discontinue a participant for safety reasons, protocol violations, or administrative necessities.

Procedures Following Discontinuation:

(1) Upon discontinuation, the investigator will document the primary reason for withdrawal.

(2) A final safety and efficacy assessment will be performed whenever feasible.

(3) All discontinued participants will be followed as per protocol, with safety monitoring continuing until any unresolved AEs are resolved or stabilized.

4.6. Termination criteria

The study may be temporarily or permanently terminated under the following circumstances:

(1) Termination triggers

1) Safety concerns:

Evidence of unacceptable risk (e.g., higher-than-expected SAEs, confirmed treatment-related deaths).

2) Futility/lack of efficacy:

Interim analysis will show statistically significant lack of benefit (predefined stopping rules).

3) Protocol violations:

Major deviations compromising scientific integrity or participant welfare.

4) Regulatory/ethical orders:

Mandated halt by:

① Ethics committee (EC);

② Regulatory authorities.

(2) Post-termination procedures

1) Participant safety:

① Conduct immediate clinical follow-up for all enrolled subjects;

② Provide alternative therapies if warranted.

2) Data preservation:

① Lock and archive all databases;

② Retain termination documentation (e.g., EC/regulatory correspondence).

3) Reporting:

① Notify all relevant parties (investigators, ECs, regulators) within 7 days;

② Submit final termination report within 30 days, including:

Reason for termination, summary of accrued data, and safety outcomes.

(3) Documentation requirements

1) Temporary vs. permanent:

Clearly label suspension duration/resumption conditions if temporary.

2) Audit trail:

Preserve unmodified records of termination decisions.

5. Treatment

5.1. Treatment protocol

Participants will be randomly assigned in a 1:1:1 ratio to one of three treatment groups: (1) The YBT group: YBT capsules will be administered orally. (2) The LOPM group: LOPM will be performed. (3) The YBT + LOPM group: Participants will receive both YBT capsules and LOPM. In addition to their assigned treatments, all participants will performe standardized lumbar functional exercises throughout the treatment period.

5.11. Description of treatment methods

(1) YBT: YBT capsules (Jiangsu Kanion Pharmaceutical Co., Ltd.; National Drug Approval No. Z20010045) were administered orally at a dose of 3 capsules (0.42 g per capsule) three times daily, preferably after meals.

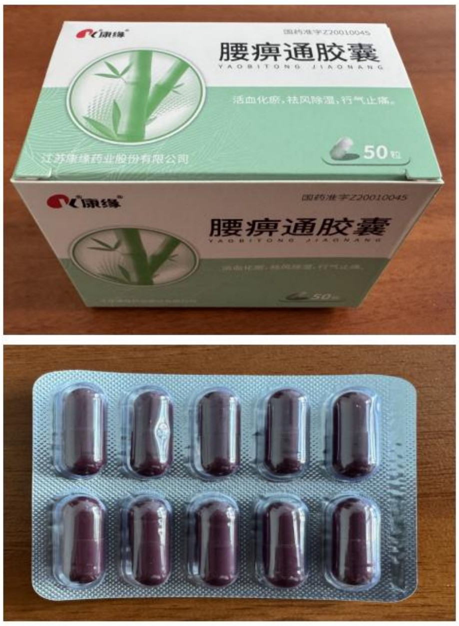

Figure 1. YBT capsules used in the trial

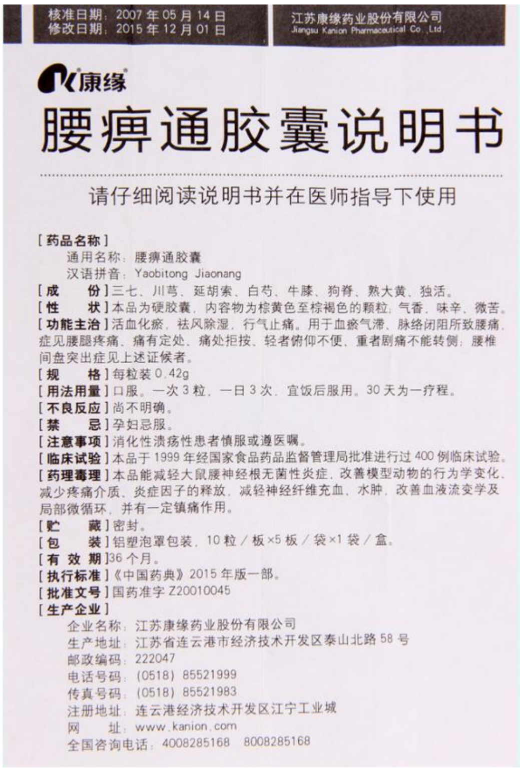

核准日期：2007 年 05 月 14 日
修改日期：2015 年 12 月 01 日
江苏康缘药业股份有限公司
Jiangsu Kanion Pharmaceutical Co.,Ltd.

康缘

腰痹通胶囊说明书

请仔细阅读说明书并在医师指导下使用

［药品名称］
通用名称：腰痹通胶囊
汉语拼音：Yaobitong Jiaonang
［成　　份］三七、川芎、延胡索、白芍、牛膝、狗脊、熟大黄、独活。
［性　　状］本品为硬胶囊，内容物为棕黄色至棕褐色的颗粒；气香，味辛、微苦。
［功能主治］活血化瘀，祛风除湿，行气止痛。用于血瘀气滞、脉络闭阻所致腰痛，症见腰腿疼痛、痛有定处、痛处拒按、轻者俯仰不便、重者剧痛不能转侧；腰椎间盘突出症见上述证候者。
［规　　格］每粒装 0.42g
［用法用量］口服。一次 3 粒，一日 3 次，宜饭后服用。30 天为一疗程。
［不良反应］尚不明确。
［禁　　忌］孕妇忌服。
［注意事项］消化性溃疡性患者慎服或遵医嘱。
［临床试验］本品于 1999 年经国家食品药品监督管理局批准进行过 400 例临床试验。
［药理毒理］本品能减轻大鼠腰神经根无菌性炎症，改善模型动物的行为学变化、减少疼痛介质、炎症因子的释放，减轻神经纤维充血、水肿，改善血液流变学及局部微循环，并有一定镇痛作用。
［贮　　藏］密封。
［包　　装］铝塑泡罩包装，10 粒／板×5 板／袋×1 袋／盒。
［有 效 期］36 个月。
［执行标准］《中国药典》2015 年版一部。
［批准文号］国药准字 Z20010045
［生产企业］
企业名称：江苏康缘药业股份有限公司
生产地址：江苏省连云港市经济技术开发区泰山北路 58 号
邮政编码：222047
电话号码：(0518) 85521999
传真号码：(0518) 85521983
注册地址：连云港经济技术开发区江宁工业城
网　　址：www.kanion.com
全国咨询电话：4008285168　8008285168

Figure 2. YBT capsules drug instruction leaflet

(2) LOPM technique

The LOPM will be performed 3 times per week. The general procedure will be as follows: First, assess the tension of the soft tissues to identify the location of the lesion. Then, apply lumbar tendon-regulation techniques, which include four methods: palm-pressing, finger-kneading, shoulder-pulling and lumbar-pushing, and leg-pulling and lumbar-pushing. Use palm-pressing and finger-kneading to relax spasming lumbar muscles, focusing on loosening local tender points. Follow this with tendon-regulation techniques such as shoulder-pulling and lumbar-pushing or leg-pulling and lumbar-pushing to fully stretch the spine and further relax the surrounding soft tissues. Next, perform the step-by-step oblique pull manipulation. Finally, conduct a quality check of the manipulation.

1) Assessment method

The patient lies prone on the bed with relaxed lumbar muscles. The practitioner stands on one side of the patient and uses the index, middle, and ring fingers to press along three lines on the lower back: the midline (spinous processes of the lumbar vertebrae) and the bilateral paravertebral lines (small articular processes on either side of the midline). Check the position of each spinous process along the midline, then palpate along the paravertebral lines to assess muscle tension, nodules, or cord-like lesions. Identify tenderness or radiating pain at the affected vertebral segments. Next, perform percussion with the palm heel from top to bottom, comparing both sides to determine the location of the lesion.

2) Tendon-regulation techniques

① Palm-pressing method:

Place overlapping palms with the heel of the hand along the side of the lumbar spinous processes. Apply gradual pressure from top to bottom toward the opposite side, transmitting force from the shoulder through the forearm to the contact area, targeting the vertebral lamina and interlaminar spaces. Repeat 5 times for approximately 6 minutes.

② Finger-kneading method: On identified tender points, use the thumb to perform gentle, rhythmic, small-amplitude kneading. Then, maintain steady pressure while performing additional techniques such as pressing, rotating, or transverse/longitudinal plucking. The force should be light and pliable until tenderness in the affected area diminishes. Duration: about 6 minutes.

③ Shoulder-pulling and lumbar-pushing method: For example, to pull the patient's right shoulder and push the lumbar region, the practitioner stands on the patient's left side. The left hand is placed on the front of the patient's shoulder joint, while the right palm heel presses from the opposite side of the thoracic spinous process downward to the L3 spinous process as the shoulder is pulled backward. Duration: 30 seconds per side, 1 minute total for both sides.

④ Leg-pulling and lumbar-pushing method: For example, to pull the patient's right leg and push the lumbar region, the practitioner stands on the patient's left side. The left palm heel is placed on the affected lumbosacral area, while the right hand holds the patient's leg 3 cm above the knee. As the leg is pulled backward, the left hand rhythmically pushes and fixes the affected area. Duration: 30 seconds per side, 1 minute total for both sides.

3) Step-by-step oblique pull manipulation

① Preparation: The patient lies on their side with the body level. The upper leg is fully flexed at the knee and hip, while the lower leg remains straight. The practitioner stands facing the patient beside the bed and places fingers on the spinous processes above and below the affected segment. Instruct the patient to use these points as pivots, arch the back, rotate the shoulder backward, and the hip forward to actively rotate the lumbar region to its maximum range.

② Locking: The practitioner places both elbows (inner side of the upper forearms) against the patient's front shoulder and back hip. The elbow at the hip remains fixed, while the elbow at the shoulder gently pushes the shoulder backward until a distinct fixation sensation is achieved. Then, the practitioner's elbows apply opposite-direction elastic pushes (at approximately 45° to the torso axis) 2–3 times to complete the locking.

③ Pulling: Once the patient is fully relaxed, the practitioner uses their waist to drive both elbows and delivers a follow-through force to complete the oblique pull (amplitude of 3–5°). An audible click may occur but should not be forced.

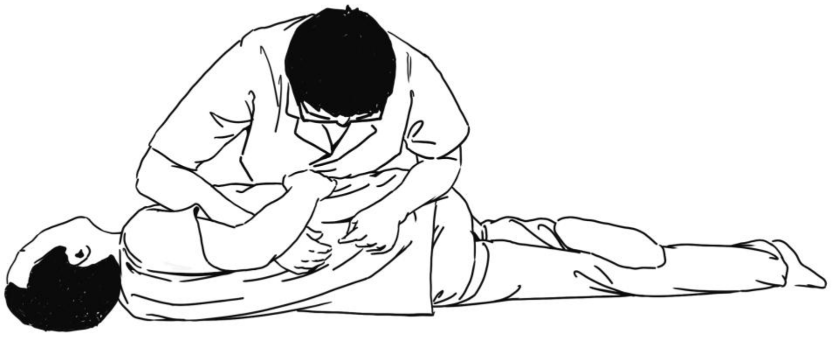

Figure 3. LOPM technique

4) Quality check of LOPM

After the manipulation, check the following to verify the therapeutic effect:

① Reduced muscle tension: If the technique is applied correctly, the initially high tension in the bilateral lumbar muscles should decrease.

② Palpation check: With the patient prone and hands at their sides, the practitioner gently presses the soft tissues on both sides of the affected spinous process with the thumb and middle finger. The cord-like tension in the muscles should diminish, and tenderness should lessen.

③ Functional improvement: After standing, the patient should report relief in lumbar and leg symptoms, improved range of motion, and reduced pain if the manipulation was effective.

5) Training and assessment for LOPM technique

The training and assessment for the LOPM technique will be uniformly organized by Wangjing Hospital, China Academy of Chinese Medical Sciences. Training will be conducted by physicians with over 20 years of hands-on experience in the LOPM technique. Assessment will be carried out upon completion of training, and only those who meet the required standards will be permitted to perform the technique in research settings.

LOPM technique assessment score sheet

Trainee name: Assessment date: Institution:	
**Relaxation technique assessment items**	**Scoring**
Operator's posture standardization	□Excellent (5) □Good (4) □Pass (3) □Fail (2)
Movement coordination	□Excellent (5) □Good (4) □Pass (3) □Fail (2)
Procedural logic	□Excellent (10) □Good (8) □Pass (6) □Fail (4)
Patient comfort level	□Excellent (10) □Good (8) □Pass (6) □Fail (4)
Completion of technique	□Excellent (10) □Good (8) □Pass (6) □Fail (4)
**Oblique manipulation technique assessment**	**Scoring**
Standardization of procedure	□Excellent (20) □Good (15) □Pass (10) □Fail (5)
Completion of key techniques	□Excellent (20) □Good (15) □Pass (10) □Fail (5)

Qualified outcome	☐Excellent (20) ☐Good (15) ☐Pass (10) ☐Fail (5)
**Instructions**:<br>**Excellent:** All operations fully comply with standards, with significant therapeutic outcomes.<br>**Good:** Basically meets standards, with good effectiveness.<br>**Pass:** Minor deficiencies exist, but overall performance is qualified.<br>**Fail:** Non-standard operation or results fail to meet expected outcomes.<br>**A score of 80 or above is considered qualified-eligible.**	
**Total score:**	**Assessment results**:<br>**☐Qualified-eligible for clinical trial participation**<br>**☐Not qualified-requires retraining**
**Trainee's signature:**<br>**Date:**	**Assessor's signature:**<br>**Date:**

(3) Lumbar functional exercise routine

The lumbar functional exercise routine will be performed twice daily (once in the morning and once in the afternoon), consisting of five movements: rotational stretch, swallow dive, supine bridge, knee-to-chest curl, and air cycling. Each session will last 10 minutes.

1) Rotational stretch:

Stand with feet shoulder-width apart, arms relaxed at sides. Inhale, then raise arms: left arm bends across the chest (palm down), right arm extends sideways (palm down). Slowly rotate the torso to the right, eyes following the right hand. Hold for 3–5 seconds at maximum rotation, then return to upright. Repeat symmetrically to the left. Repeat 6–8 times.

Key points: Maintain steady posture; avoid rapid movements; keep feet stationary.

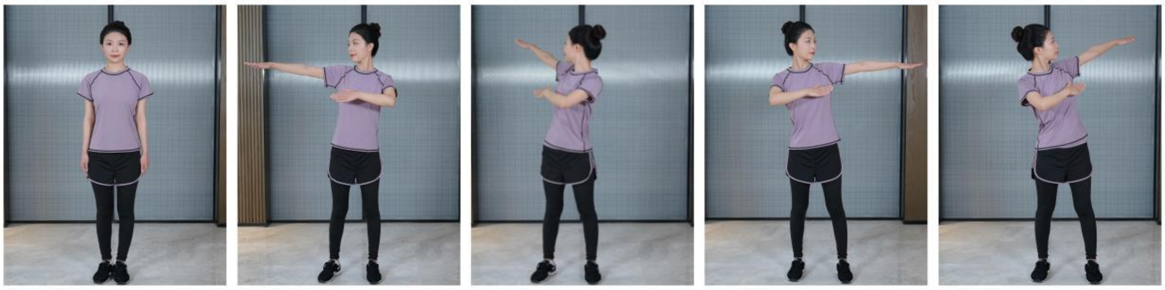

Figure 4. Rotational stretch

2) Swallow dive:

Lie prone with arms at sides and legs straight. Inhale, then slowly lift the head, arms, and legs (keep elbows/knees straight) into a “flying swallow” position. Hold for 3–5 seconds, exhale while lowering, then relax for 5 seconds. Repeat 6–8 times.

Key points: Look forward; avoid hyperextending the neck; adjust range per ability.

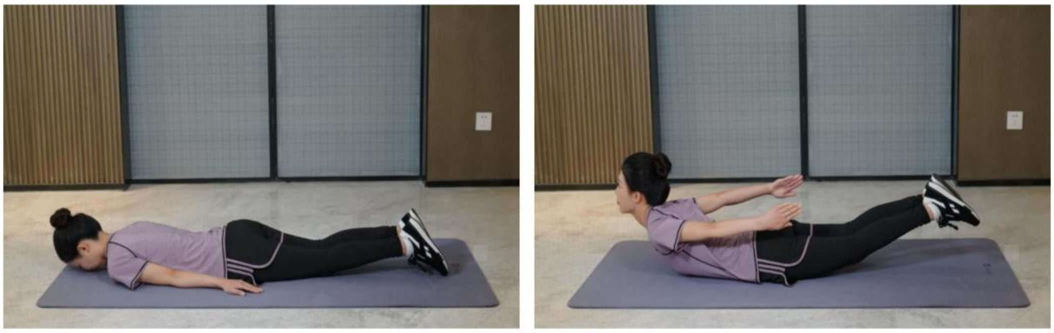

Figure 5. Swallow dive

3) Supine bridge:

Lie supine with knees bent heels/elbows/shoulders as support. Lift the pelvis until the abdomen aligns with the knees. Hold for 3–5 seconds. Exhale while lowering slowly, then relax for 5 seconds. Repeat 6–8 times.

Key points: Control movement speed; adapt range to fitness level.

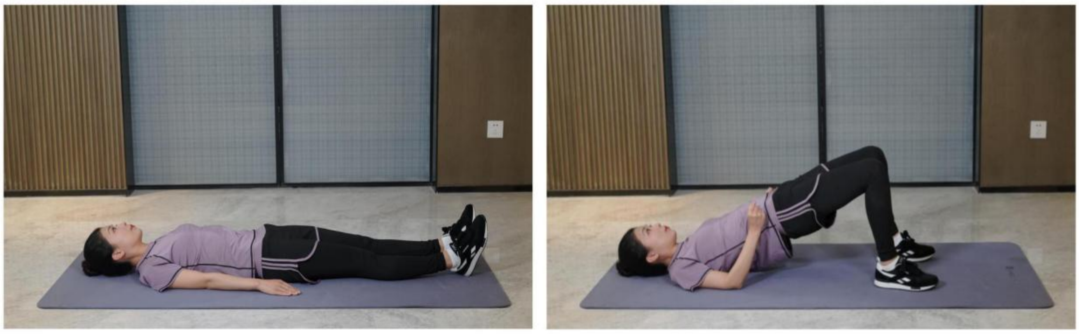

Figure 6. Supine bridge

4) Knee-to-chest curl:

Lie supine, bend knees toward the chest, and hug them (or hold thighs if needed). Curl into a tight ball, lower back pressed to the bed. Hold for 3–5 seconds. Exhale while releasing, then relax for 5 seconds. Repeat 6–8 times.

Key points: Move smoothly; avoid jerking.

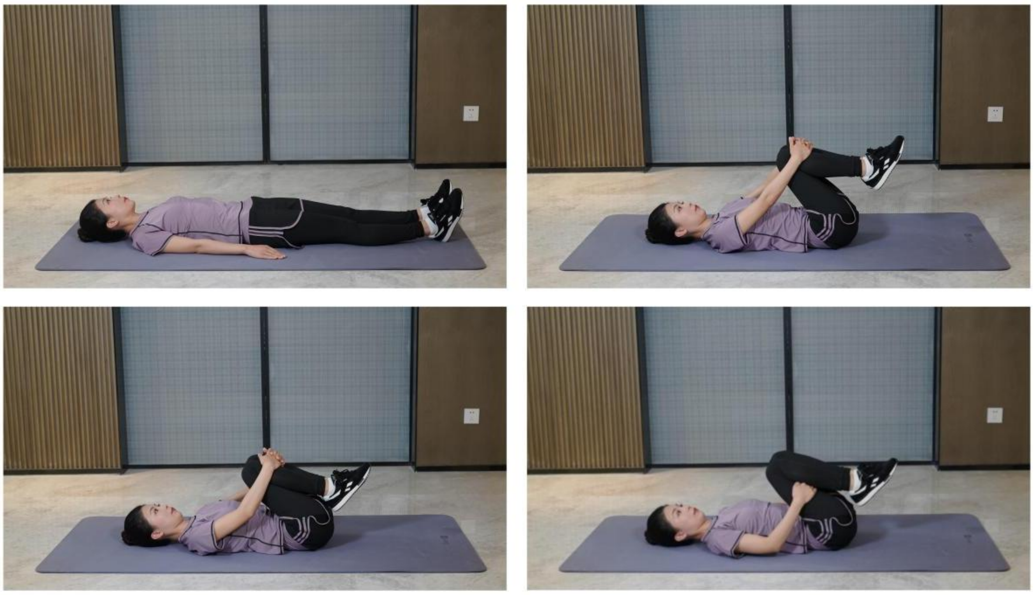

Figure 7. Knee-to-chest curl

5) Air cycling:

Lie supine with arms at sides. Bend hips/knees and simulate cycling motions alternately. Repeat 6–8 times.

Key points: Stabilize the torso; maintain moderate speed.

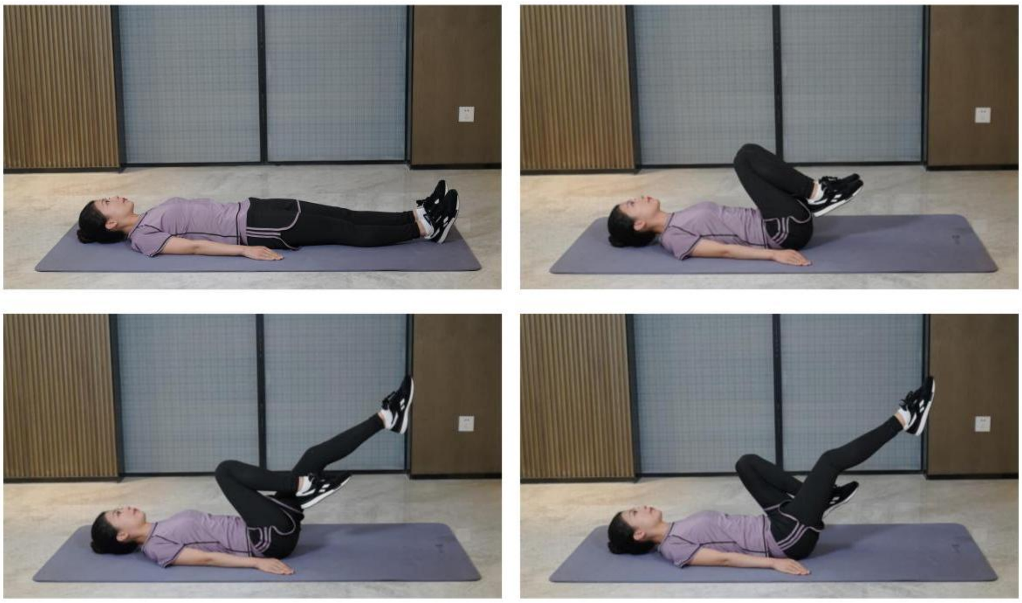

Figure 8. Air cycling

6) Cool-down:

After the routine, rest supine for 1–2 minutes while breathing deeply to relax.

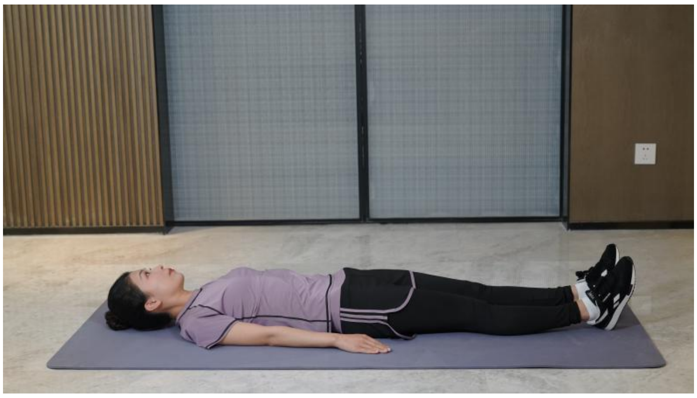

Figure 9. Cool-down

The research team will develop standardized lumbar functional exercise instruction manuals, which will be distributed to participants upon enrollment. Additionally, study physicians will provide in-person training to ensure participants master the exercise techniques.

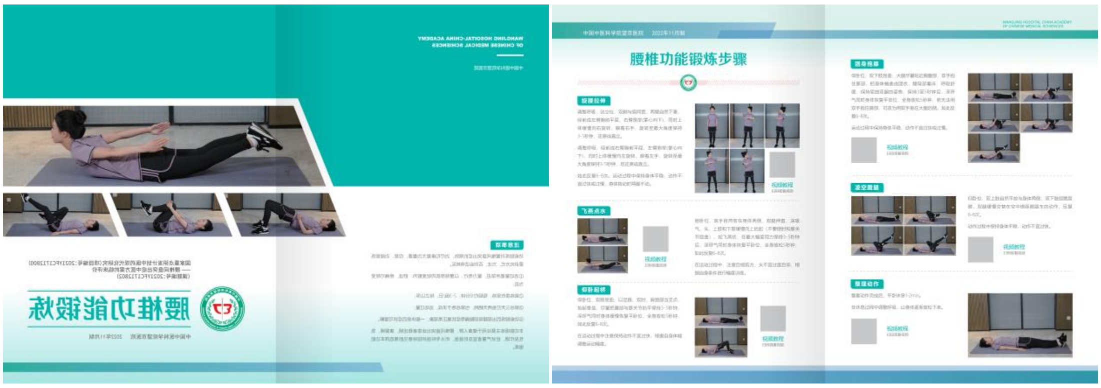

Figure 10. Lumbar functional exercise instruction manual

5.2. Concomitant medication protocol

To ensure participant safety and minimize confounding factors, rescue medications will be permitted in cases of intolerable pain, with all usage documented in CRFs.

5.3. Drug dispensing and storage protocol

(1) Drug receipt & documentation

1) Upon receiving trial medications, each study site will complete a drug receipt form, signed by two authorized personnel.

2) The form will be duplicated, with one copy retained by the lead institution and the other by the local trial site.

(2) Storage conditions

1) Medications will be stored at room temperature, protected from light and moisture.

2) Each site will designate a responsible staff member for drug custody.

3) All trial drugs shall be secured in a locked dedicated cabinet.

(3) Dispensing & tracking

1) Medications will be allocated sequentially based on patient enrollment order, with each package assigned a unique sequential ID number.

2) All drug dispensing and returns will be promptly recorded in a dedicated log.

(4) Drug return & accountability

1) At study conclusion, all unused medications and empty packaging will be retrieved.

2) A drug return form will be signed by both the site and sponsor.

3) Residual drugs will be returned to the lead investigational unit for proper disposal.

5.4. Investigational drug management

(1) Centralized drug control

The PI’s institution (Wangjing Hospital, China Academy of Chinese Medical Sciences) will be responsible for coordinating with pharmaceutical manufacturers and maintaining unified control over drug batch numbers.

(2) Prohibitions

The investigational drugs will be strictly prohibited from being sold or used for commercial purposes.

(3) Drug accountability records

Comprehensive documentation will be maintained for all aspects of drug handling, including:

1) Quantity tracking.

2) Transportation logs.

3) Delivery records.

4) Receipt verification.

5) Distribution details.

6) Return and destruction of unused medications.

(4) Investigator responsibilities

Investigators will ensure that:

1) All investigational drugs are used exclusively for study participants.

2) Dosage and administration strictly adhere to the study protocol.

3) Any unused medications are returned to the PI's institution (Wangjing Hospital, China Academy of Chinese Medical Sciences);

4) Drug management is assigned to specifically designated personnel.

5) Proper records are maintained to prevent diversion to non-study individuals.

(5) Monitoring and oversight

The entire drug management process, including:

1) Supply chain.

2) Usage.

3) Storage conditions.

4) Disposition of unused medications.

Will be subject to regular audits by monitors from the PI's institution (Wangjing Hospital, China Academy of Chinese Medical Sciences), with all audit records properly maintained.

5.5. Quality control (QC) and monitoring of investigational drugs

5.5.1. Drug QC

(1) All investigational drugs will be required to meet predefined quality standards, including:

1) Manufacturing batch records.

2) Expiration dates.

3) Storage conditions (temperature, humidity, light protection).

(2) Regular quality inspections will be conducted by designated QC personnel, with results documented in the trial master file.

(3) Any quality deviations (e.g., compromised integrity, storage violations) will be:

1) Reported immediately to the sponsor and regulatory authorities (if required);

2) Quarantined until resolution.

3) Replaced if non-compliant.

5.5.2. Expiry date management of investigational drugs

(1) Strict monitoring of expiration dates for all investigational drugs will be implemented.

(2) Key requirements:

1) All drugs will be used within their valid expiry period.

2) Expired drugs shall be:

① Immediately labeled as “EXPIRED-DO NOT USE”;

② Promptly removed from investigational stock;

③ Prohibited from use in clinical research.

3) Expiration dates will be clearly documented in:

① Drug receipt records;

② Drug dispensing logs;

③ All relevant usage documentation.

5.6. Adverse drug reactions (ADRs) and reporting

(1) ADR Reporting

1) All suspected ADRs related to the investigational drug(s) will be:

① Promptly documented in the CRF.

② Reported to the Institutional Review Board (IRB)/EC if deemed clinically significant.

③ Submitted to regulatory authorities (per local requirements) for serious/life-threatening reactions.

2) Subject withdrawal due to ADRs:

① Detailed documentation will be required (onset, severity, outcome, causality assessment).

② Appropriate medical intervention will be provided.

③ Follow-up will continue until resolution/stabilization.

(2) Drug safety monitoring

1) Proactive surveillance for drug-related AEs:

① Regular patient health assessments (scheduled and as-needed) will be conducted.

② Protocol-defined safety labs/vital signs will be monitored.

2) Serious adverse events (SAEs):

① Immediate reporting (<24 hrs.) to sponsor and IRB will be implemented.

② Expedited regulatory reporting will be submitted if applicable.

③ Corrective actions: Dose modification/treatment discontinuation + supportive care will be taken.

5.7. Emergency drug management protocol

(1) Handling emergencies

In the event of emergency situations (e.g., compromised storage conditions, expired drugs), immediate action will be taken, including but not limited to:

1) Reallocation or replacement of affected drugs.

2) Safe disposal of non-compliant medications.

Full documentation of emergency actions will be maintained to ensure audit transparency.

(2) Drug shortage contingency plan

If investigational drug shortages occur during the study:

1) Immediate notification to the drug supplier/manufacturer will be made.

2) Expedited resupply procedures will be implemented to ensure continuity.

3) Protocol-compliant adjustments to drug allocation (e.g., prioritization of high-risk participants) will be executed.

4) Regulatory compliance will be maintained throughout.

6. Observation endpoints and clinical outcomes

6.1. Clinical observation endpoints

Efficacy measures will be recorded at baseline and 3 days, 1 week, 2 weeks, 6 weeks, 14 weeks, and 26 weeks after randomization. The measurement instruments will be ODI for the functional disability, VAS for the leg and low back pain, and 12-Item Short Form Health Survey (SF-12)[11] for the quality of life. Blood routine tests and liver and kidney function tests will be conducted before randomization and at 2 weeks after randomization. In addition, detailed records of each patient’s gastrointestinal discomfort, skin

allergies, and other adverse events will be collected at 2 weeks after randomization to assess safety. At the 26-week post-randomization follow-up, a repeat MRI will be performed. The change in the sagittal area of the disc herniation from baseline to 26 weeks will be measured, with a reduction of ≥40% defined as the occurrence of disc resorption. The disc resorption rates will be calculated. The primary outcome will be change in ODI from baseline to week 2. The minimal clinically important difference (MCID) for ODI will be set at ≥7 points, consistent with prior studies. Secondary outcomes will include leg and low back pain VAS, SF-12, adverse events, and disc resorption rates.

(1) ODI

ODI assesses the effect of pain on normal daily activity including the ability to and intensity of lifting, care for oneself, walk, sit, sexual function, stand, social life, sleep and travel, ranging from 0 (no disability) to 100 (maximum disability possible).

Modified ODI

Pain intensity	I have no pain now.	☐
	The pain is very mild now.	☐
	The pain is moderate now.	☐
	The pain is fairly severe now.	☐
	The pain is very severe now.	☐
	The pain is the worst imaginable now.	☐
Personal care	I can look after myself normally without causing extra pain.	☐
	I can look after myself normally, but it causes extra pain.	☐
	It is painful to look after myself, and I am slow and careful.	☐
	I need some help but can manage most of my personal care.	☐
	I need help every day in most aspects of self-care.	☐
	I cannot dress, wash, or stand without help.	☐
Lifting	I can lift heavy weights without extra pain.	☐
	I can lift heavy weights, but it gives extra pain.	☐
	Pain prevents me from lifting heavy weights off the floor.	☐
	Pain prevents me from lifting heavy weights, but I can manage light weights.	☐
	I can lift only very light weights.	☐
	I cannot lift or carry anything.	☐
Walking	Pain does not prevent me from walking any distance.	☐
	Pain prevents me from walking more than 1 mile.	☐
	Pain prevents me from walking more than ¼ mile.	☐
	Pain prevents me from walking more than 100 yards.	☐
	I can walk only with crutches or a stick.	☐
	I am in bed most of the time and must crawl to the toilet.	☐
Sitting	I can sit in any chair if I like.	☐
	I can sit in my favorite chair if I like.	☐

	Pain prevents me from sitting for more than 1 hour.	☐
	Pain prevents me from sitting for more than 30 minutes.	☐
	Pain prevents me from sitting for more than 10 minutes.	☐
	Pain prevents me from sitting at all.	☐
Standing	I can stand if I want without extra pain.	☐
	I can stand as long as I want but it gives me extra pain.	☐
	Pain prevents me from standing for more than 1 hour.	☐
	Pain prevents me from standing for more than 30 minutes.	☐
	Pain prevents me from standing for more than 10 minutes.	☐
	Pain prevents me from standing at all.	☐
Sleeping	My sleep is never disturbed by pain.	☐
	My sleep is occasionally disturbed by pain.	☐
	Because of pain, I have less than 6 hours of sleep.	☐
	Because of pain, I have less than 4 hours of sleep.	☐
	Because of pain, I have less than 2 hours of sleep.	☐
	Pain prevents me from sleeping at all.	☐
Social life	My social life is normal and gives me no extra pain.	☐
	My social life is normal but increases my pain.	☐
	Pain has no significant effect on my social life apart from limiting more energetic activities.	☐
	Pain has restricted my social life, and I do not go out as often.	☐
	Pain has restricted my social life to my home.	☐
	I have no social life because of pain.	☐
Traveling	I can travel anywhere without pain.	☐
	I can travel anywhere but it gives extra pain.	☐
	Pain is bad but I can manage journeys over 2 hours.	☐
	Pain restricts me to journeys of less than 1 hour.	☐
	Pain restricts me to short necessary journeys under 30 minutes.	☐
	Pain prevents me from traveling except to receive treatment.	☐
Housework/job	Normal housework/job activities do not cause any pain.	☐
	Normal housework/job activities cause pain, but I can still manage to perform them.	☐
	I can perform most housework/job activities, but pain prevents me from doing physically demanding tasks (e.g., lifting, moving objects, cleaning).	☐
	Due to pain, I can only perform light physical tasks.	☐
	Due to pain, I am unable to perform even light physical tasks.	☐
	Due to pain, I cannot perform any job or housework at all.	☐

(2) VAS

The VAS is used to assess the intensity of the patient's leg and low back pain. Specifically, a 10-cm horizontal line is presented, with the left endpoint labeled “No pain (0)” and the right endpoint labeled

"Worst pain imaginable (10)." The patient is instructed to mark a vertical line ("/") on the scale according to their perceived pain intensity. The physician then measures the distance (in centimeters) from the left endpoint to the patient's mark, with a longer distance indicating greater pain severity.

No pain (0) Worst pain imaginable (10)

(3) SF-12

The SF-12 assesses the quality of life through physical and mental dimensions, with scores typically ranging from 0 to 100. Higher scores indicate a better quality of life.

SF-12

**In general, would you say your health is:**				
□Excellent	□Very good	□Good	□Fair	□Poor

**The following questions are about activities you might do during a typical day. Does your health now limit you in these activities? If so, how much?**			
	Yes, limited a lot	Yes, limited a little	No, not limited at all
Moderate activities such as moving a table, pushing a vacuum cleaner, bowling, or playing golf.	□	□	□
Climbing several flights of stairs.	□	□	□

**During the past 4 weeks, have you had any of the following problems with your work or other regular daily activities as a result of your physical health?**		
Accomplished less than you would like.	□Yes	□No
Were limited in the kind of work or other activities.	□Yes	□No

**During the past 4 weeks, have you had any of the following problems with your work or other regular daily activities as a result of any emotional problems (such as feeling depressed or anxious)?**		
Accomplished less than you would like.	□Yes	□No
Did work or activities less carefully than usual.	□Yes	□No

**During the past 4 weeks, how much did pain interfere with your normal work (including work outside the home and housework)?**				
□Not at all	□A little bit	□Moderately	□Quite a lot	□Extremely

**These questions are about how you have been feeling during the past 4 weeks. For each question, please give the one answer that comes closest to the way you have been feeling. How much of the time during the past 4 weeks...**						
	All the time	Most of the time	A good bit of the time	Some of the time	A little of the time	None of the time
Have you felt calm & peaceful?	□	□	□	□	□	□
Did you have a lot of energy?	□	□	□	□	□	□
Have you felt downhearted and blue?	□	□	□	□	□	□

**During the past 4 weeks, how much of the time has your physical health or emotional problems interfered with your social activities (like visiting friends, relatives, etc.)?**				
□All of the time	□Most of the time	□Some of the time	□A little of the time	□None of the time

6.2. Safety assessment

Blood routine tests and liver and kidney function tests were conducted before randomization and at 2 weeks after randomization. In addition, detailed records of each patient's gastrointestinal discomfort, skin allergies, and other AEs were collected at 2 weeks after randomization to assess safety.

6.3. Disc resorption assessment

At the 26-week post-randomization follow-up, a repeat MRI will be performed. The change in the sagittal area of the disc herniation from baseline to 26 weeks will be measured, with a reduction of ≥40% defined as the occurrence of disc resorption. The disc resorption rates will be calculated.

The maximum area of each disc herniation was measured on sagittal T2-weighted MRI sequences using Image J software (National Institutes of Health, Bethesda, MD, USA), with tracings made from the posterior edge of adjacent vertebrae in accordance with the measurement standards and definitions established by the North American Spine Society (NASS), the American Society of Spine Radiology (ASSR), and the American Society of Neuroradiology (ASNR) task force.[12] All measurements were independently performed by two spine surgeons with extensive experience, and the average of their measurements was taken as the final result. The reliability assessment demonstrated good to excellent reproducibility.

The following table summarizes the data collection schedule and key assessment time points:

Study schedule

**Phase**	**Study visit**	**Treatment period**			**Follow-up period**		
**Visit**	Enrollment	Visit 1	Visit 2	Visit 3	Visit 4	Visit 5	Visit 6
**Timing**	Day 0	Day 3	Week 1	Week 2	Week 6	Week 14	Week 26
**Baseline data**							
Inclusion criteria confirmation	√						
Exclusion criteria confirmation	√						
Informed consent obtained	√						
Demographic data collected	√						
**Efficacy assessments**							
ODI	√	√	√	√	√	√	√
Leg pain VAS	√	√	√	√	√	√	√
Low back pain VAS	√	√	√	√	√	√	√
SF-12	√	√	√	√	√	√	√
**Safety monitoring**							
Complete blood count	√			√			
Liver function tests	√			√			
Renal function tests	√			√			

Adverse events recording		√	√	√			
**Imaging**							
X-ray	√						
CT	√						
MRI	√						√

7. AEs observation and analysis

An AE will refer to any untoward medical occurrence in a patient administered a pharmaceutical product or medical intervention, which does not necessarily have a causal relationship with the treatment. AEs will encompass abnormal laboratory findings, ADRs, anticipated adverse effects of the investigational therapy, and events where causality assessment will be required. All AEs will be managed and reported in accordance with established regulatory and ethical guidelines.

7.1. Criteria for AE assessment

Common AEs will include:

(1) LOPM-related: Worsening low back pain, restricted lumbar mobility, aggravated lower limb numbness or pain.

(2) Drug-related: Gastrointestinal reactions (e.g., constipation, epigastric pain, nausea, vomiting), allergic reactions (e.g., skin rash, urticaria), and other side effects such as physical dependence or drug tolerance.

SAEs will be defined as progressively worsening symptoms, including:

(1) Muscle weakness.

(2) Cauda equina syndrome.

(3) Severe intractable pain.

7.2. Analysis of causality and severity

All AEs will be systematically documented, and their association with the treatment will be assessed. Causality will be classified according to the Guidelines for Clinical Research of New Traditional Chinese Medicine Drugs (Trial) into five categories:

(1) Definite.

(2) Probable.

(3) Possible.

(4) Unlikely.

(5) Unrelated.

Severity will be graded as follows:

(1) Mild: No significant impact on daily activities (work or study).

(2) Moderate: Interference with daily activities.

(3) Severe: Marked limitation of daily activities.

All AEs will be recorded along with the corresponding management measures and treatment outcomes.

7.3. Documentation of Common AEs

All AEs will be recorded following each treatment session. Particular attention will be given to:

(1) LOPM-related AEs (e.g., symptom exacerbation).

(2) Drug-related AEs (e.g., gastrointestinal disturbances, cutaneous allergic reactions).

(3) Other potential treatment-emergent AEs, which will be actively solicited and documented.

For each AE, the following details will be collected:

(1) Clinical manifestations.

(2) Severity (mild/moderate/severe).

(3) Onset date.

(4) Duration.

(5) Resolution date.

(6) Interventions applied.

(7) Assessed causality to treatment (definite/probable/possible/unlikely/unrelated);

(8) Outcome.

A comprehensive analysis will be performed, accounting for comorbidities and concomitant therapies, to evaluate their association with the study treatment. These data will be used to systematically characterize:

(1) Complications.

(2) Contraindications.

(3) Indications.

7.4. Management of AEs

(1) LOPM-related AEs

A small subset of patients may experience worsening lumbar symptoms or reduced mobility during LOPM, typically due to excessive force or abrupt manipulation irritating nerve roots. Recommended management will include:

1) Immediate rest in a supine position until symptom relief.

2) Lumbar brace support during ambulation post-recovery.

3) Analgesics (oral or intravenous) if symptoms persist, with resolution typically within 24 – 48 hours.

(2) Drug-related AEs:

Gastrointestinal reactions (e.g., epigastric pain, nausea, constipation, vomiting), allergic reactions, or dependence/tolerance may occur. Management follows:

1) Reassessment of drug continuation based on causality.

2) Discontinuation if warranted, with documented follow-up to monitor outcomes.

(3) SAEs

For progressive symptoms (e.g., muscle weakness, cauda equina syndrome, or intractable pain):

1) Immediate clinical evaluation by a physician will be conducted.

2) Surgical intervention if clinically indicated will be conducted.

(4) Protocol for AE Reporting

All AEs will require:

1) Prompt protective measures by investigators.

2) Notification of the IRB and lead research unit within 24 hours.

3) Signed, dated reports submitted per regulatory standards.

7.5. Analysis of AEs

All cases of study withdrawal or treatment discontinuation due to AEs will be systematically documented. Data will include:

(1) Demographics.

(2) AE characteristics.

(3) Timing relative to treatment.

(4) Interventions attempted.

(5) Final outcome.

7.6. Abnormal laboratory findings

Investigators will evaluate the clinical significance of abnormal laboratory results and provide potential explanations. Abnormal laboratory findings attributable to previously reported AEs will be documented as part of the original AE record.

Independent AE reporting will be required for clinically significant abnormal laboratory results (excluding those linked to known AEs) meeting ≥1 of the following criteria:

(1) Associated clinical symptoms (e.g., jaundice with elevated bilirubin).

(2) Lead to adjustment of the investigational treatment (dose modification/discontinuation).

(3) Require intervention (e.g., concomitant medication changes or additional therapies).

8. QC and quality assurance

8.1. Analysis of study influencing factors

The following factors will be rigorously controlled to ensure study validity and minimize bias:

(1) Patient enrollment

Strict adherence to inclusion/exclusion criteria will be enforced through:

1) Protocol training for all investigators.

2) Centralized screening audits (10% random checks).

(2) Standardization of LOPM

1) Operator qualification: All therapists will hold attending physician or higher credentials.

2) Competency assurance: Mandatory training and certification by the study team prior to participation

will be required.

(3) Lumbar rehabilitation guidance

Patients will receive:

1) Standardized exercise instruction (in-person demonstration).

2) Supplementary materials: Video tutorials and illustrated manuals (free access).

(4) Blinded outcome assessment

1) Role separation: Therapists and evaluators will be distinct personnel.

2) Blinding protocol: Evaluators will be masked to treatment allocation during assessments.

(5) Data entry quality

Dual-process verification:

1) Two dedicated staff per site will perform alternate data entry.

2) All entries will require double-confirmation before locking.

8.2. Infrastructure for QC

To ensure data integrity and study compliance, this trial will establish a dedicated research laboratory, equipped with:

(1) Secure data management systems

1) Password-protected computers with encrypted storage for electronic CRFs and source documents.

2) Backup servers with daily automated updates to prevent data loss.

(2) Standardized hardware

1) Site-specific workstations configured with identical software to minimize operational variability.

2) High-resolution scanners for digitizing paper-based records (e.g., signed consent forms).

(3) Remote monitoring capacity

VPN-enabled devices for real-time access by auditors/regulators, ensuring compliance with GCP on-site inspection equivalency.

8.3. Study training and monitoring protocols

To ensure rigorous adherence to study protocols and data accuracy, the following training and quality monitoring systems will be implemented:

(1) Three-tier QC system

1) First-level (site-level): Daily checks by research assistants for CRF completeness.

2) Second-level (center-level): Weekly audits by designated quality inspectors.

3) Third-level (study-wide): Monthly independent monitoring by external auditors.

(2) Research record verification

1) Dual independent data entry: Two research assistants will separately transcribe paper-based records into electronic CRFs, followed by consistency validation via electronic checks.

2) Real-time error correction: Discrepancies will be resolved through online source data verification with

timestamped corrections.

(3) Weekly patient management review

Key variables will be audited:

1) Subject ID accuracy.

2) Treatment completion status.

3) Investigator signatures.

4) Enrollment dates.

Data reporting plans will be updated weekly based on findings.

(4) Quality inspection procedures

Checklist-driven audits (weekly minimum) will cover:

1) Source documentation.

2) AE reporting compliance.

3) Investigational product accountability.

PI oversight: PI will review and sign all audit reports, with corrective actions documented.

(5) Monitoring plan

Bimonthly on-site monitoring: Two independent monitors will verify:

1) 100% source data against electronic CRFs for critical fields.

2) Protocol deviations and resolution logs.

Cross-center consistency checks will ensure uniform implementation.

8.4. Strategies to improve participant compliance

(1) Importance of compliance

Adherence monitoring: Participants will be required to attend all scheduled treatments, with protocol deviations (e.g., missed visits) documented, including:

1) Reasons for non-compliance (e.g., logistical barriers, side effects);

2) Corrective actions taken (e.g., rescheduling, re-education).

Patient education: Will emphasized the impact of consistent participation on treatment efficacy and safety outcomes.

(2) Common causes of non-adherence

Will be identified through pre-trial surveys and interim interviews:

1) Perceived lack of efficacy.

2) Misconceptions about recovery timelines.

3) Low trust in therapeutic protocols.

(3) Compliance optimization measures

1) Enhanced communication

Pre-treatment counseling: Will include detailed discussions about:

① Individualized treatment goals,

② Expected therapeutic trajectories.

2) Proactive follow-up

Structured phone calls at mid-treatment intervals will be used to:

① Address concerns (e.g., “Why hasn’t my pain improved?”).

② Reinforce protocol importance.

8.5. QC and quality assurance system

(1) Primary-level (Site) quality checks

1) QC officer appointment: Each sub-center will appoint a qualified quality control officer responsible for weekly audits.

2) Checklist-based verification: The QC officer will evaluate:

① Source data documentation (e.g., CRFs, medical records).

② Investigational product accountability;

③ AE reporting compliance.

3) PI oversight: The PI will review and sign all QC reports weekly, with corrective actions documented for identified issues.

(2) Secondary-level (central) monitoring

1) Monitoring plan

① On-site visits: Will be conducted monthly by independent monitors, with frequency adjusted based on study progress/risk.

② 100% source data verification: Critical fields will be cross-checked against original records.

2) Monitoring procedures

Scope: will cover:

① Protocol adherence (e.g., informed consent, eligibility criteria);

② Drug dispensing logs;

③ AE/SAE handling timelines.

Monitor qualifications:

① Medical/clinical research background;

② Certified in GCP and electronic data capture (EDC) system operation.

On-site audits: Will Verify:

① Participant identity (e.g., ID checks).

② Data authenticity (e.g., lab reports, signed consents).

Electronic CRF Validation: Will ensure concordance between electronic CRFs and source documents.

Logic checks: Will include ad hoc reviews for implausible data (e.g., outliers in lab values).

Reporting: Post-visit reports will include:

① Findings;

② Actionable recommendations;

③ Resolution timelines.

(3) Tertiary-level (Regulatory) audits

The study team will facilitate external audits by higher-tier institutions (e.g., ECs, funding agencies), providing:

1) Unrestricted access to source data.

2) Documentation of all QC/monitoring activities.

9. Data administration

9.1. Source data documentation

Source data will encompass all original records generated during the clinical study, including:

(1) Clinical source documents:

1) Medical charts.

2) Laboratory/test reports.

3) Pharmacy dispensing logs.

4) Signed informed consent forms.

(2) Derived study records:

1) Completed CRFs.

2) Data clarification forms.

3) Statistical analysis reports with raw datasets.

Requirements:

(1) Retention: Will be stored for ≥5 years post-trial completion.

(2) Accessibility: Must be available for regulatory inspection/auditing.

(3) Verification: 100% source data verification will be performed for critical efficacy/safety endpoints.

9.2. Data recording and reporting standards

Timeliness:

(1) All source data must be transcribed into CRFs within 24 hours of collection.

(2) Electronic CRF submission via web-based EDC systems within 72 hours, with automated timestamping.

Quality standards:

(1) Entries must be contemporaneous, legible, and complete (no undocumented corrections).

(2) Dual verification: Critical fields (e.g., primary endpoints, SAEs) will require independent review by a second investigator.

System oversight:

Data managers (QC team) will perform:

(1) Real-time validation (range/consistency checks).

(2) Query resolution for discrepancies (e.g., missing/outlier data).

(3) Database locking after final audit.

9.3. Source document archiving standards

(1) Storage protocols

1) Active studies: Source documents will be maintained in secure, access-controlled research offices at each clinical site.

2) Completed studies: Documents will be transferred within 30 days to a centralized archive with:

① Climate-controlled environments (temperature/humidity monitoring),

② Fireproof/waterproof storage systems.

(2) Access security

1) Role-based permissions:

① Investigators: Will have read/write access during active trials.

② Monitors/auditors: Will have read-only access for verification.

③ Third parties: Will require IRB approval for access.

2) Electronic records: Will be protected via two-factor authentication and encrypted backups.

(3) Retention period

1) Minimum duration: 5 years after study completion, or longer if required by:

① Local regulations (e.g., China's Regulations on Human Biomedical Research).

② Sponsor/funding agency policies.

2) Destruction protocol: Documents will be shredded/electronically erased with witnessed documentation.

9.4. Source document access regulations

(1) General access policy

Access to source documents must comply with:

1) Institutional policies of the hosting hospital.

2) Archival regulations of the clinical trial unit.

(2) On-site monitoring procedures

1) Facilitated access: Clinical sites must provide immediate, unhindered access to source documents for monitors during scheduled visits.

2) Designated review area: Inspection will occur in a secured, monitored space to ensure:

① Document integrity (no unauthorized removal);

② Patient confidentiality (no public exposure).

(3) Copying of source documents

1) Redaction requirement: All copies must have personally identifiable information removed, including:

① Names;

② ID numbers;

③ Contact details.

2) Authorization: Copies will require written approval from the PI and IRB.

9.5. Data verification protocol

(1) Electronic data review process

Monitors must:

1) Access the monitor interface of the EDC system.

2) Verify 100% of critical fields (e.g., primary endpoints, SAEs) against source documents (e.g., medical records, lab reports).

3) Mark each data field as "reviewed" in the EDC system, with automated timestamps.

(2) Discrepancy management

1) Query initiation: If inconsistencies are identified, monitors must:

① Generate electronic queries (e.g., via EDC or dedicated data clarification forms).

② Specify the exact variance.

2) Resolution tracking:

① Site investigators will respond within 72 hours with corrections/justifications.

② All queries will be closed-loop documented in the EDC audit trail.

9.6. CRF completion guidelines

(1) Completion protocol

1) Exclusive responsibility: CRFs must be filled directly by investigators (not delegated to non-clinical staff).

2) Real-time documentation: Data must be recorded during or immediately after patient visits to ensure:

① Timeliness (≤24 hours post-encounter);

② Accuracy (no retrospective entries);

③ Completeness (all protocol-required fields).

(2) Correction standards

1) Error modification: Any changes will require:

① Single strikethrough of original entry (no obscuring);

② Legible annotation of corrected value;

③ Investigator’s signature + date;

④ Brief rationale.

2) Prohibition: No erasures, white-out, or overwriting will be permitted.

(3) Quality assurance

1) Dual review:

① Site PIs will verify 100% of CRFs for:

Protocol compliance and consistency with source documents.

② Central monitor will audit 10% randomly (higher risk for critical fields).

2) Electronic backups: Scanned CRFs will be archived in read-only PDF/A format.

9.7. Electronic data reporting protocol

(1) Roles and Responsibilities

Electronic CRF entry personnel:

1) Each study site must appoint two trained electronic CRF entry specialists responsible for:

① Reviewing source documents (e.g., research charts) for completeness and accuracy.

② Entering data into the electronic CRF system.

2) Qualifications:

① Certified in good clinical practice (GCP);

② Completed protocol-specific electronic CRF training.

(2) Data flow process

1) Investigator documentation:

After each patient visit, the investigator must complete the research chart by end-of-day and hand it to the electronic CRF entry team.

2) Electronic CRF entry and validation:

① Primary entry: The first electronic CRF specialist will enter data into the system within 24 hours.

② Secondary verification: The second specialist will independently:

Cross-checks 100% of entries against source documents and resolve system-generated queries (e.g., missing data, outliers).

(3) Correction and audit trail

1) Error handling:

① Any corrections must be annotated with a reason (e.g., "Transcription error");

② Signed/dated by both the investigator and electronic CRF specialist.

2) Audit trail:

The electronic CRF system will log all modifications with:

① Timestamp;

② User ID;

③ Justification for changes.

9.8. Database review and locking procedures

(1) Data cleaning & query resolution

Data manager responsibilities:

1) Review all resolved queries from data clarification forms and update the database accordingly.

2) Issue new data resolution queries if inconsistencies persist.

3) Document all changes in the audit trail, including:

① Investigator's response;

② Date of resolution;

③ Data manager's verification signature.

(2) Database locking process

1) Pre-lock checks:

① 100% verification of critical variables (primary/secondary endpoints, SAEs).

② Blinded review: An independent statistician will confirm data integrity without access to treatment allocation.

2) Final lock approval:

① PIs and statistician will sign off on the data quality assurance report.

② Database will be permanently locked with write-access disabled.

3) Statistical handoff:

① Read-only copy will be transferred to the biostatistics team.

② Metadata + audit logs will be archived separately for regulatory inspection.

10. Statistical analysis

An independent third party (Institute of Basic Research in Clinical Medicine, China Academy of Chinese Medical Sciences) will perform all statistical analyses. The primary analysis will follow the ITT principle, including all randomly assigned patients. A supporting per-protocol (PP) analysis will be conducted to assess the robustness of the primary findings.

10.1 Basis for protocol development

This study protocol of statistical analysis has will be developed in accordance with the following guidelines and requirements:

(1) China's Guidelines for Biostatistics in Clinical Trials.

(2) China's GCP guidelines.

(3) The Technical Guidelines for Pharmaceutical Research in Various Stages of New Traditional Chinese Drug Development (Trial) issued in 2020.

(4) The specific project requirements of the National Key Research and Development Program of China.

10.2 Analysis populations

10.3 Safety analysis population

The safety analysis set will include all randomized subjects who received at least one time of the assigned study intervention. All safety evaluations-including the recording, documentation, and analysis of any AEs or reactions-will be performed on this population.

10.4 ITT

The ITT population will include all randomized participants. The primary analyses will be based on the ITT principle to preserve the unbiased nature of the randomization and to provide a conservative estimate of the treatment effect under real-world clinical conditions.

10.5 PP

The PP population will be required to satisfy all the following criteria:

(1) Are correctly randomized and receive at least one time of the study intervention.

(2) Have no major protocol deviations (pre-specified) that could substantially impact the efficacy evaluation (e.g., major violations of inclusion/exclusion criteria discovered post-randomization, use of prohibited concomitant therapies, or compliance below a pre-defined threshold).

(3) Complete the assessment for the primary efficacy endpoint.

10.6 Statistical method

The primary efficacy analysis will follow the intention-to-treat (ITT) principle, including all randomly assigned patients. All analyses will be performed using R software, version 4.4.0 (R Foundation for Statistical Computing). Missing data for the longitudinal outcomes will be handled under the assumption of missing at random using multiple imputation. Imputation will be performed using the Multivariate Imputation by Chained Equations (MICE) package in R, generating 5 imputed datasets. The imputation model will include all longitudinal outcome measures from all-time points and all baseline covariates listed above. Results from the imputed datasets will be pooled according to Rubin’s rules. Baseline characteristics will be summarized using descriptive statistics. For the continuous outcomes, between-group differences at each specific time point and within-group changes from baseline will be evaluated via pairwise comparisons of the estimated marginal means derived from the linear mixed model (LMM), which will be fitted using the lme4 package (version 1.1.35). These comparisons will be conducted using the emmeans package (version 1.10.0) with Tukey’s method for adjustment of P values to control the

family-wise error rate. The model will include fixed effects for treatment group, time (modeled as a categorical factor), and their interaction. The model will be adjusted for the following prespecified baseline covariates: sex, age, body mass index, total duration of lumbar disc disease, duration of the current progressive episode, and degree of herniation at the symptomatic disc level. For categorical outcomes, between-group comparisons will be performed using the chi-square test or Fisher's exact test as appropriate, while the Bonferroni correction will be applied to adjust the significance level and control for type I error arising from multiple comparisons. All statistical tests will be two-sided, with a P value of less than .05 considered statistically significant. Institute of Basic Research in Clinical Medicine, China Academy of Chinese Medical Sciences will perform all statistical analyses.

11. Ethical issues

11.1. Ethic review

This study will follow the Declaration of Helsinki and Chinese regulations for clinical trials. Before starting, the study protocol will be reviewed and approved by EC at each study site. The comments of EC may include agreement, agreement after necessary revision, disagreement, and termination or suspension of the approved study.

11.2. Ethical review system

(1) Ethical oversight structure

1) Lead EC

① Role: Will review the scientific validity and ethical compliance of the study protocol.

② Output: Will issue approval documents disseminated to:

PIs at all participating sites, local ECs, and QC team.

2) Local institutional ECs

① Role: Will assess site-specific feasibility, including:

Investigator qualifications and facility/infrastructure adequacy.

② Output: Approval documents will be forwarded to: Lead PI, lead EC, and QC team.

(2) Authority and limitations

1) No protocol modifications: Local ECs cannot alter core elements (e.g., drug dosage, inclusion/exclusion criteria).

2) Study suspension power: Local ECs must halt trials if ethical violations are identified.

(3) AE Governance

1) SAE review: Local ECs must convene within 48 hours of SAE occurrence.

2) Cross-institution notification: Conclusions will be shared with lead EC and all participating site ECs.

(4) Protocol amendments

1) Initiation: Local ECs may request amendments only to enhance participant protection.

2) Process: Requires:

① Written justification;

② Lead EC approval;

③ Uniform implementation across all sites.

(5) Inter-EC communication

1) Mandatory channels: Secure email/encrypted platforms for:

① Expedited SAE reporting;

② Amendment requests.

2) Response time: ≤72 hours for critical issues.

11.3. Benefits and risks assessment

11.3.1 Potential benefits

Participants may derive the following direct benefits from this study:

(1) Enhanced medical care:

1) Standardized diagnostic evaluations.

2) Protocol-defined therapeutic interventions.

(2) Clinical improvement:

Potential alleviation of LDHR symptoms (e.g., pain, mobility).

Note: Benefits are neither guaranteed nor the primary study objective.

11.3.2 Anticipated risks

(1) Treatment-related risks:

1) LOPM:

① Transient exacerbation of lumbar pain;

② Increased lower limb numbness/radicular pain.

2) Pharmacotherapy:

① Gastrointestinal events (e.g., nausea, constipation);

② Allergic dermatitis.

(2) Disease progression risks:

1) Failure to achieve symptom relief.

2) Rare but severe events (e.g., cauda equina syndrome).

11.3.3 Risk mitigation strategies

(1) Pre-screening: Exclusion of high-risk participants (e.g., spinal instability).

(2) Real-time monitoring: Protocol-defined thresholds for therapy discontinuation.

(3) SAE protocols: Immediate surgical referral for neurological deficits.

11.4. Informed consent process

11.4.1 Pre-enrollment requirements

(1) Comprehensive disclosure:

1) Investigators must provide written and verbal explanations (in lay language) covering:

① Study purpose and duration;

② Potential benefits and risks;

③ Alternative treatment options.

2) Use approved consent documents (vetted by the EC).

(2) Participant rights:

1) Emphasize the right to withdraw at any time without penalty.

2) Clarify voluntary participation (no coercion).

(3) Documentation:

1) Signed informed consent forms must be obtained prior to any study procedures.

2) Store original informed consent forms in secure, access-controlled archives.

11.4.2 Special circumstances

(1) Legally authorized representatives: Will be permitted for cognitively impaired participants.

(2) Re-consent: Will be required for substantial protocol amendments affecting risks/benefits.

11.5. Participant recruitment procedures

11.5.1 Recruitment methods

(1) On-Site advertising:

Posters will be displayed in clinics/hospitals of participating centers, including:

1) Study title/purpose.

2) Eligibility criteria (age, diagnosis, etc.).

3) Contact information.

(2) Digital outreach:

WeChat (and other approved social media platforms) will be used to disseminate:

1) EC-approved recruitment notices.

2) Pre-screening questionnaires (to assess preliminary eligibility).

11.5.2 Eligibility verification

Two-stage screening:

(1) Initial screening: Will be conducted by research coordinators via phone/online forms.

(2) In-person confirmation: Full assessment against inclusion/exclusion criteria by investigators.

11.5.3 Ethical compliance

(1) Ad content review: All materials will be pre-approved by the EC to avoid undue influence.

(2) No coercive language: Prohibited phrases (e.g., “Guaranteed cure”).

11.6. Participant rights and protections

11.6.1 Voluntary participation & withdrawal rights

(1) Voluntary consent:

Participation is entirely voluntary, with no impact on:

1) Clinical care quality.

2) Patient-provider relationship.

3) Access to alternative treatments.

(2) Unpenalized withdrawal:

1) Participants may withdraw at any time without justification.

2) Standard-of-care therapies will remain available.

11.6.2 Medical record handling

(1) Documentation standards:

1) All research data (e.g., CRFs, lab reports) will be integrated into hospital medical records.

2) Original research documents will be archived securely.

(2) Access permissions:

1) Authorized access will be limited to:

① Study investigators;

② Regulatory inspectors;

③ ECs.

2) De-identification: Published data will exclude personally identifiable information.

11.6.3 Privacy safeguards

(1) Legal compliance: Will adherence to China’s Personal Information Protection Law.

(2) Data anonymization: Research datasets will use unique participant codes.

11.7. EC continuing review

11.7.1 Scope and frequency of review

The EC must conduct continuing reviews of all approved studies at least annually, covering:

(1) Protocol amendments:

1) Assess scientific/ethical validity of proposed changes (e.g., new risks, procedures).

2) Verify re-consent requirements for ongoing participants.

(2) SAEs:

1) Review aggregated SAE reports to evaluate risk-benefit balance.

2) Require immediate ad-hoc reviews for unexpected fatal/life-threatening SAEs.

(3) Annual progress reports:

Evaluate:

1) Enrollment rates.

2) Withdrawal reasons.

3) Interim safety data.

11.7.2 Review procedures

(1) Full-committee review for:

1) First renewal.

2) High-risk amendments (e.g., dose escalation).

(2) Expedited review for minor changes (e.g., typographical corrections).

(3) Documentation:

All reviews will generate dated approval certificates or modification requests.

11.7.3 Regulatory compliance

(1) Deadlines: Annual reviews must occur ±30 days of approval anniversary.

(2) Non-compliance: Studies will lapse if renewal is overdue by >60 days.

11.8. EC inspection procedures

11.8.1 Roles and responsibilities

During regulatory inspections or audits of the EC, the following personnel must comply with institutional Standard Operating Procedures:

(1) EC office secretary:

1) Prepare documentation: Organize and provide:

① Approved study protocols and amendments;

② Meeting minutes and voting records;

③ SAE review reports;

④ Continuing review files.

2) Coordinate logistics: Schedule interviews, arrange meeting spaces.

(2) EC members:

1) Participate in interviews: Explain deliberation processes for:

① Initial approvals;

② Risk-benefit assessments;

③ Controversial decisions.

2) Defend ethical oversight.

(3) EC chairperson:

Lead inspections: Present overall EC governance, including:

1) Conflict-of-interest management.

2) Training records of members.

3) Annual performance self-assessments.

11.8.2 Inspection readiness

(1) Pre-inspection:

1) Conduct mock audits biannually using checklists.

2) Ensure 72-hour document retrieval capacity.

(2) During inspection:

Provide unedited, timestamped records (no post-hoc modifications).

(3) Post-inspection:

Submit corrective action plans within 15 working days for identified deficiencies.

12. Storage and archiving of research materials

(1) Active study phase

1) Storage location: All source documents and data must be kept in a dedicated, access-controlled research office at each study site.

2) Custodian: Will be managed by the site research assistant under the PI's supervision.

3) Access restrictions:

① Only authorized personnel (e.g., investigators, monitors) may handle materials.

② No unauthorized disclosure or removal will be permitted without written approval from the lead study unit.

(2) Post-study archiving

1) Centralized transfer: Within 30 days of study completion, all materials must be:

① Cataloged (e.g., indexed by participant ID/visit date).

② Securely packaged;

③ Transferred to the lead institution's archive.

2) Retention requirements:

① Will be retained for ≥5 years post-study, or longer if required by local regulations.

② Electronic data will be archived in read-only formats (e.g., PDF) with encrypted backups.

(3) Confidentiality safeguards

1) Anonymization: All shared datasets must remove personally identifiable information.

2) Destruction protocol: Post-retention, materials will be:

① Shredded (paper);

② Digitally erased (electronic) with certification.

13. Clinical summary

After the completion of statistical analyses, the statistical unit will release the statistical report with a seal.

14. Research participating institutions and task allocation

14.1 Allocation of research tasks

Institutions	Allocation of research tasks	Principal investigator
Wangjing Hospital, China Academy of Chinese Medical Sciences	Research design, equipment preparation, clinical research and project summary	Liguo Zhu
Affiliated Hospital of Changchun University of Chinese Medicine	Clinical research	Shaojun Li
Affiliated Hospital of Shaanxi University of Chinese Medicine	Clinical research	Puwei Yuan
Affiliated Hospital of Shandong First Medical University	Clinical research	Bin Shi
Dongzhimen Hospital, Beijing University of Chinese Medicine	Clinical research	Yusong Jia
First Teaching Hospital of Tianjin University of Traditional Chinese Medicine	Clinical research	Ping Wang
Gansu Provincial Hospital of Traditional Chinese Medicine	Clinical research	Jirong Zhao
Guangdong Provincial Hospital of Chinese Medicine	Clinical research	Bolai Chen
Liaoning University of Traditional Chinese Medicine	Clinical research	Xuefeng Guan
Luoyang Orthopedic-Traumatological Hospital of Henan Province	Clinical research	Jiayi Guo
Nanfang Hospital, Southern Medical University	Clinical research	Yikai Li
Suzhou Hospital of Traditional Chinese Medicine	Clinical research	Hong Jiang
The First Hospital of Hunan University of Chinese Medicine	Clinical research	Shaofeng Yang
Jiangsu Kanion Pharmaceutical Co., Ltd	Pharmaceutical preparation and adverse event consultation	Liang Li

14.2 Allocation of enrolled patients

Clinical Center	Number of Patients
Wangjing Hospital, China Academy of Chinese Medical Sciences	Total n = 42 (14 per group)
Affiliated Hospital of Changchun University of Chinese Medicine	Total n = 33 (11 per group)
Affiliated Hospital of Shaanxi University of Chinese Medicine	Total n = 33 (11 per group)
Affiliated Hospital of Shandong First Medical University	Total n = 33 (11 per group)
Dongzhimen Hospital, Beijing University of Chinese Medicine	Total n = 33 (11 per group)

The National Key Research and Development Program of China, No. 2021YFC1712802

First Teaching Hospital of Tianjin University of Traditional Chinese Medicine	Total n = 33 (11 per group)
Gansu Provincial Hospital of Traditional Chinese Medicine	Total n = 33 (11 per group)
Guangdong Provincial Hospital of Chinese Medicine	Total n = 33 (11 per group)
Liaoning University of Traditional Chinese Medicine	Total n = 33 (11 per group)
Luoyang Orthopedic-Traumatological Hospital of Henan Province	Total n = 33 (11 per group)
Nanfang Hospital, Southern Medical University	Total n = 33 (11 per group)
Suzhou Hospital of Traditional Chinese Medicine	Total n = 33 (11 per group)
The First Hospital of Hunan University of Chinese Medicine	Total n = 33 (11 per group)

15. References

1. Knezevic NN, Candido KD, Vlaeyen JWS, et al. Low back pain. Lancet. 2021 Jul 3;398(10294):78-92. doi: 10.1016/S0140-6736(21)00733-9.

2. GBD 2017 Disease and Injury Incidence and Prevalence Collaborators. Global, regional, and national incidence, prevalence, and years lived with disability for 354 diseases and injuries for 195 countries and territories, 1990-2017: a systematic analysis for the Global Burden of Disease Study 2017. Lancet. 2018 Nov 10;392(10159):1789-1858. doi: 10.1016/S0140-6736(18)32279-7.

3. Omidi-Kashani F, Hejrati H, Ariamanesh S. Ten Important Tips in Treating a Patient with Lumbar Disc Herniation. Asian Spine J. 2016 Oct;10(5):955-963. doi: 10.4184/asj.2016.10.5.955.

4. Altun I, Yüksel KZ. Lumbar herniated disc: spontaneous regression. Korean J Pain. 2017 Jan;30(1):44-50. doi: 10.3344/kjp.2017.30.1.44.

5. Deyo RA, Mirza SK. CLINICAL PRACTICE. Herniated Lumbar Intervertebral Disk. N Engl J Med. 2016 May 5;374(18):1763-72. doi: 10.1056/NEJMcp1512658.

6. Fairbank JC, Pynsent PB. The Oswestry Disability Index. Spine (Phila Pa 1976). 2000 Nov 15;25(22):2940-52; discussion 2952. doi: 10.1097/00007632-200011150-00017.

7. Goldberg H, Firtch W, Tyburski M, et al. Oral steroids for acute radiculopathy due to a herniated lumbar disk: a randomized clinical trial. JAMA. 2015;313(19):1915-1923.

8. Kreiner DS, Hwang SW, Easa JE, et al. An evidence-based clinical guideline for the diagnosis and treatment of lumbar disc herniation with radiculopathy. Spine J. 2014 Jan;14(1):180-91. doi: 10.1016/j.spinee.2013.08.003.

9. Chinese Orthopaedic Association. Clinical guidelines for the diagnosis and treatment of lumbar disc herniation. Chin J Orthop. 2020;40(8):477-487. doi:10.3760/cma.j.cn121113-20200402-00213.

10. Huskisson EC. Measurement of pain. Lancet. 1974 Nov 9;2(7889):1127-31. doi: 10.1016/s0140-6736(74)90884-8.

11. Ware J Jr, Kosinski M, Keller SD. A 12-Item Short-Form Health Survey: construction of scales and preliminary tests of reliability and validity. Med Care. 1996 Mar;34(3):220-33. doi: 10.1097/00005650-199603000-00003.

12. Fardon DF, Williams AL, Dohring EJ, et al. Lumbar disc nomenclature: version 2.0: recommendations of the combined task forces of the North American Spine Society, the American Society of Spine Radiology, and the American Society of Neuroradiology. Spine (Phila Pa 1976). 2014 Nov 15;39(24): E1448-65. doi: 10.1097/BRS.0b013e3182a8866d.
