# Supplementary material for: Combination of Yaobitong capsules and lumbar oblique pull manipulation for moderate pain in lumbar disc herniation with radiculopathy: a multicenter, randomized, three-arm, parallel-group controlled trial
Source: Front Neurol. 2026 Jun 26;17:1853703. doi: 10.3389/fneur.2026.1853703 (PMC13349874; doi:10.3389/fneur.2026.1853703)
Supplement: Supplementary file 2 [file Supplementary_file_2.docx]

**eTable 1. Demographics and Baseline Characteristics of Study Participants**

| **Characteristic** | **YBT (n=146)** | **LOPM (n=137)** | **YBT + LOPM (n=143)** |
| --- | --- | --- | --- |
| Age, mean (SD), y | 43.6 (12.8) | 45.8 (14.4) | 45.4 (15.1) |
| Sex, No. (%) |  | | |
| Female | 75 (51.4) | 91 (66.4) | 82 (57.3) |
| Male | 71 (48.6) | 46 (33.6) | 61 (42.7) |
| BMI, mean (SD) | 24.4 (4.4) | 23.8 (3.3) | 24.1 (3.8) |
| Symptomatic herniated disc level, No. (%) |  |  |  |
| L1-L2 | 1 (0.7) | 1 (0.7) | 2 (1.4) |
| L2-L3 | 8 (5.5) | 6 (4.4) | 5 (3.5) |
| L3-L4 | 16 (11.0) | 12 (8.8) | 15 (10.5) |
| L4-L5 | 84 (57.5) | 83 (60.6) | 85 (59.4) |
| L5-S1 | 37 (25.3) | 35 (25.5) | 36 (25.2) |
| Degree of herniation at the symptomatic disc level^a^, No. (%) |  | | |
| Bulge | 31 (21.3) | 36 (26.3) | 36 (25.2) |
| Protrusion | 104 (71.2) | 95 (69.3) | 102 (71.3) |
| Extrusion | 11 (7.5) | 6 (4.4) | 5 (3.5) |
| Nature of work, No. (%) |  | | |
| Mental labor | 56 (38.4) | 51 (37.2) | 60 (41.9) |
| Physical labor | 31 (21.2) | 25 (18.2) | 24 (16.8) |
| Physical and mental work | 35 (24.0) | 32 (23.4) | 34 (23.8) |
| Unemployed | 10 (6.8) | 9 (6.6) | 8 (5.6) |
| Retired | 14 (9.6) | 20 (14.6) | 17 (11.9) |
| Income level, No. (%) |  | | |
| ＜CNY 3,000 | 42 (28.8) | 35 (25.5) | 35 (24.5) |
| CNY 3,000-10,000 | 77 (52.7) | 79 (57.7) | 79 (55.2) |
| CNY 10,000-20,000 | 23 (15.8) | 20 (14.6) | 25 (17.5) |
| ＞CNY 20,000 | 4 (2.7) | 3 (2.2) | 4 (2.8) |
| Duration of disease, mean (SD), d | 948.6 (1200.9) | 1232.9 (1515.5) | 1484.0 (2021.4) |
| Duration of disease progression, mean (SD), d | 40.4 (93.7) | 58.9 (168.0) | 60.1 (181.9) |
| History of lumbar spine trauma, No. (%) | 4 (2.7) | 2 (1.5) | 5 (3.5) |
| Comorbidity, No. (%) |  | | |
| Hypertension | 12 (8.2) | 15 (10.9) | 15 (10.5) |
| Type-II diabetes | 5 (3.4) | 5 (3.6) | 2 (1.4) |
| Coronary heart disease | 1 (0.7) | 1 (0.7) | 1 (0.7) |
| Hyperlipidemia | 7 (4.8) | 4 (2.9) | 5 (3.5) |
| Osteoporosis | 1 (0.7) | 0 (0.0) | 2 (1.4) |
| Cervical spondylosis | 20 (13.7) | 15 (10.9) | 16 (11.2) |
| Knee osteoarthritis | 6 (4.1) | 5 (3.6) | 3 (2.1) |
| ODI^b^, mean (SD) | 37.6 (15.3) | 38.4 (15.8) | 38.8 (16.3) |
| Leg pain VAS score^c^, mean (SD) | 4.5 (1.7) | 4.6 (1.5) | 4.8 (1.7) |
| Low back pain VAS score^c^, mean (SD) | 5.1 (1.2) | 5.0 (1.2) | 5.1 (1.1) |
| SF-12 PCS^d^, mean (SD) | 35.6 (7.4) | 35.8 (6.5) | 36.5 (7.6) |
| SF-12 MCS^d^, mean (SD) | 45.7 (8.5) | 45.0 (7.8) | 46.4 (7.9) |

Abbreviations: BMI, body mass index; CNY, China Yuan; LOPM, lumbar oblique pull manipulation; MCS, Mental Component Summary; ODI, Oswestry Disability Index; PCS, Physical Component Summary; SD, standard deviation; SF-12, 12-Item Short Form Health Survey; VAS, visual analog scale; YBT, Yaobitong.

^a^ Herniation degree was classified on axial and sagittal T2-weighted magnetic resonance imaging (MRI) sequences according to widely accepted morphological criteria: Bulge (circumferential extension >50% of disc circumference); Protrusion (focal herniation with the base wider than the apex); and Extrusion (herniation with the apex wider than the base or the presence of a sequestered fragment).

^b^ ODI assesses the effect of pain on normal daily activity including the ability to and intensity of lifting, care for oneself, walk, sit, sexual function, stand, social life, sleep and travel, ranging from 0 (no disability) to 100 (maximum disability possible).

^c^ Scores for leg pain and low back pain could range from 0 (no pain) to 10 (pain as bad as you can imagine).

^d^ SF-12 assesses the quality of life through physical and mental dimensions, with scores typically ranging from 0 to 100. Higher scores indicate a better quality of life.

**eTable 2. Outcome Measures in the Intention-to-Treat Population**

|  | **Mean (95%CI)** | | | **Changes from baseline to each observation time point，mean (95%CI) / *p* value** | | | | | | **Difference between arms，mean (95%CI) / p value** | | | |
| --- | --- | --- | --- | --- | --- | --- | --- | --- | --- | --- | --- | --- | --- |
| **Follow up time** | **YBT**  **(n=146)** | **LOPM**  **(n=137)** | **YBT + LOPM**  **(n=143)** | **YBT** | | **LOPM** | | **YBT + LOPM** | | **YBT and YBT + LOPM** | | **LOPM and YBT + LOPM** | |
| **ODI^a^** |  |  |  |  |  |  |  |  |  |  |  |  |  |
| Day 0 | 37.6  (35.1 to 40.1) | 38.4  (35.7 to 41.1) | 38.8  (36.1 to 41.5) |  |  |  |  |  |  | -1.2  (-4.0 to 3.3) | .44 | -0.4  (-2.1 to 5.1) | .97 |
| Day 3 | 30.6  (28.3 to 32.8) | 31.5  (29.0 to 34.0) | 30.2  (27.5 to 33.0) | -7.0  (-8.9 to -5.1) | <.001 | -6.9  (-8.9 to -4.9) | <.001 | -8.5  (-10.5 to -6.6) | <.001 | 0.3  (-2.3 to 4.9) | .97 | 1.3  (-2.0 to 5.2) | .68 |
| Week 1 | 25.4  (23.3 to 27.6) | 26.1  (23.8 to 28.3) | 24.2  (21.8 to 26.5) | -12.2  (-14.1 to -10.2) | <.001 | -12.3  (-14.3 to -10.4) | <.001 | -14.6  (-16.5 to -12.7) | <.001 | 1.3  (-1.7 to 5.6) | .91 | 1.9  (-2.3 to 4.9) | .42 |
| Week 2 | 18.2  (16.2 to 20.1) | 19.0  (16.9 to 21.1) | 17.7  (15.6 to 19.8) | -19.4  (-21.4 to -17.5) | <.001 | -19.4  (-21.4 to -17.5) | <.001 | -21.1  (-23.0 to -19.1) | <.001 | 0.5  (-2.3 to 4.9) | .99 | 1.3  (-2.1 to 5.1) | .68 |
| Week 6 | 17.7  (15.7 to 19.7) | 17.8  (15.9 to 19.6) | 16.5  (14.6 to 18.3) | -19.9  (-21.8 to -18.0) | <.001 | -20.6  (-22.6 to -18.7) | <.001 | -22.3  (-24.3 to -20.4) | <.001 | 1.2  (-2.3 to 5.0) | .93 | 1.3  (-2.8 to 4.4) | .66 |
| Week 14 | 16.0  (13.9 to 18.0) | 16.5  (14.6 to 18.4) | 15.4  (13.6 to 17.2) | -21.6  (-23.5 to -19.7) | <.001 | -21.9  (-23.9 to -19.9) | <.001 | -23.4  (-25.3 to -21.4) | <.001 | 0.6  (-2.5 to 4.7) | .99 | 1.1  (-2.4 to 4.8) | .75 |
| Week 26 | 13.0  (11.3 to 14.7) | 13.4  (11.6 to 15.1) | 12.2  (10.5 to 14.0) | -24.6  (-26.5 to -22.7) | <.001 | -25.1  (-27.0 to -23.1) | <.001 | -26.5  (-28.5 to -24.6) | <.001 | 0.8  (-2.5 to 4.8) | .99 | 1.1  (-2.5 to 4.7) | .74 |
| **Leg pain VAS score^b^** | | | | | | | | | | | | | |
| Day 0 | 4.5  (4.3 to 4.8) | 4.6  (4.3 to 4.8) | 4.8  (4.5 to 5.1) |  |  |  |  |  |  | -0.3  (-0.7 to 0.2) | .17 | -0.3  (-0.4 to 0.5) | .37 |
| Day 3 | 3.9  (3.6 to 4.2) | 3.8  (3.5 to 4.0) | 4.2  (3.9 to 4.4) | -0.7  (-0.9 to -0.4) | <.001 | -0.8  (-1.0 to -0.6) | <.001 | -0.7  (-0.9 to -0.5) | <.001 | -0.3  (-0.8 to 0.1) | .17 | -0.4  (-0.5 to 0.4) | .12 |
| Week 1 | 3.2  (3.0 to 3.5) | 3.2  (2.9 to 3.4) | 3.4  (3.2 to 3.6) | -1.3  (-1.5 to -1.1) | <.001 | -1.4  (-1.6 to -1.1) | <.001 | -1.4  (-1.6 to -1.2) | <.001 | 0.2  (-0.7 to 0.2) | .42 | -0.2  (-0.4 to 0.5) | .48 |
| Week 2 | 2.4  (2.1 to 2.7) | 2.4  (2.1 to 2.7) | 2.5  (2.2 to 2.8) | -2.2  (-2.4 to -2.0) | <.001 | -2.2  (-2.4 to -1.9) | <.001 | -2.3  (-2.5 to -2.1) | <.001 | -0.1  (-0.5 to 0.3) | .55 | -0.1  (-0.3 to 0.5) | .86 |
| Week 6 | 2.3  (2.0 to 2.6) | 2.4  (2.2 to 2.7) | 2.5  (2.3 to 2.8) | -2.2  (-2.5 to -2.0) | <.001 | -2.1  (-2.4 to -1.9) | <.001 | -2.3  (-2.5 to -2.1) | <.001 | -0.2  (-0.5 to 0.4) | .34 | -0.1  (-0.3 to 0.6) | .92 |
| Week 14 | 2.1  (1.8 to 2.3) | 2.1  (1.9 to 2.4) | 2.1  (1.8 to 2.3) | -2.5  (-2.7 to -2.3) | <.001 | -2.4  (-2.7 to -2.2) | <.001 | -2.7  (-3.0 to -2.5) | <.001 | 0.0  (-0.4 to 0.5) | .93 | 0.0  (-0.3 to 0.6) | .96 |
| Week 26 | 1.8  (1.5 to 2.1) | 1.8  (1.5 to 2.0) | 1.8  (1.6 to 2.0) | -2.7  (-3.0 to -2.5) | <.001 | -2.8  (-3.0 to -2.6) | <.001 | -3.0  (-3.2 to -2.8) | <.001 | 0.0  (-0.5 to 0.4) | .91 | -0.1  (-0.4 to 0.5) | .95 |
| **Low back pain VAS score^b^** | | | | | | | | | | | | | |
| Day 0 | 5.1  (4.9 to 5.3) | 5.0  (4.8 to 5.2) | 5.1  (4.9 to 5.3) |  |  |  |  |  |  | 0.0  (-0.5 to 0.3) | .93 | -0.1  (-0.5 to 0.3) | .76 |
| Day 3 | 4.4  (4.2 to 4.6) | 4.2  (4.0 to 4.4) | 4.3  (4.1 to 4.5) | -0.7  (-0.9 to -0.5) | <.001 | -0.8  (-1.0 to -0.6) | <.001 | -0.8  (-1.0 to -0.6) | <.001 | 0.1  (-0.5 to 0.3) | .96 | -0.1  (-0.5 to 0.3) | .86 |
| Week 1 | 3.6  (3.3 to 3.8) | 3.5  (3.2 to 3.7) | 3.5  (3.3 to 3.8) | -1.5  (-1.7 to -1.3) | <.001 | -1.5  (-1.7 to -1.3) | <.001 | -1.6  (-1.8 to -1.4) | <.001 | 0.1  (-0.4 to 0.4) | .99 | 0.0  (-0.5 to 0.3) | .97 |
| Week 2 | 2.6  (2.3 to 2.9) | 2.6  (2.3 to 2.9) | 2.6  (2.3 to 2.8) | -2.5  (-2.7 to -2.3) | <.001 | -2.4  (-2.6 to -2.2) | <.001 | -2.6  (-2.8 to -2.4) | <.001 | 0.1  (-0.4 to 0.4) | .98 | 0.1  (-0.4 to 0.4) | .97 |
| Week 6 | 2.7  (2.4 to 2.9) | 2.6  (2.3 to 2.8) | 2.5  (2.3 to 2.8) | -2.4  (-2.6 to -2.2) | <.001 | -2.4  (-2.7 to -2.2) | <.001 | -2.6  (-2.8 to -2.4) | <.001 | 0.2  (-0.4 to 0.4) | .72 | 0.1  (-0.5 to 0.3) | .96 |
| Week 14 | 2.4  (2.2 to 2.7) | 2.2  (2.0 to 2.4) | 2.2  (1.9 to 2.4) | -2.7  (-2.9 to -2.5) | <.001 | -2.8  (-3.0 to -2.6) | <.001 | -3.0  (-3.2 to -2.7) | <.001 | 0.3  (-0.4 to 0.4) | .36 | 0.1  (-0.6 to 0.2) | .98 |
| Week 26 | 2.0  (1.8 to 2.2) | 1.9  (1.7 to 2.2) | 1.9  (1.6 to 2.1) | -3.1  (-3.3 to -2.9) | <.001 | -3.1  (-3.3 to -2.9) | <.001 | -3.3  (-3.5 to -3.1) | <.001 | 0.1  (-0.3 to 0.5) | .73 | 0.1  (-0.5 to 0.4) | .88 |
| **SF-12 PCS^c^** | | | | | | | | | | | | | |
| Day 0 | 35.7  (34.4 to 36.9) | 35.8  (34.7 to 36.9) | 36.5  (35.2 to 37.8) |  |  |  |  |  |  | -0.9  (-2.7 to 1.4) | .55 | -0.7  (-1.8 to 2.3) | .72 |
| Day 3 | 36.8  (35.6 to 38.0) | 37.6  (36.4 to 38.7) | 38.0  (36.7 to 39.3) | 1.1  (0.0 to 2.2) | .049 | 1.7  (0.6 to 2.9) | .003 | 1.5  (0.4 to 2.6) | .01 | -1.2  (-2.5 to 1.6) | .31 | -0.4  (-1.2 to 2.9) | .88 |
| Week 1 | 38.4  (37.2 to 39.6) | 39.2  (38.1 to 40.3) | 40.0  (38.8 to 41.2) | 2.7  (1.6 to 3.9) | <.001 | 3.4  (2.2 to 4.5) | <.001 | 3.5  (2.4 to 4.6) | <.001 | -1.6  (-2.9 to 1.2) | .13 | -0.8  (-1.2 to 2.9) | .61 |
| Week 2 | 40.9  (39.6 to 42.2) | 41.7  (40.6 to 42.8) | 41.9  (40.6 to 43.2) | 5.2  (4.1 to 6.4) | <.001 | 5.9  (4.7 to 7.0) | <.001 | 5.4  (4.3 to 6.5) | <.001 | -1.0  (-2.2 to 1.9) | .44 | -0.2  (-1.2 to 2.9) | .98 |
| Week 6 | 43.1  (41.9 to 44.3) | 43.4  (42.3 to 44.6) | 43.0  (41.7 to 44.2) | 7.5  (6.3 to 8.6) | <.001 | 7.6  (6.5 to 8.8) | <.001 | 6.5  (5.3 to 7.6) | <.001 | 0.2  (-1.6 to 2.5) | .99 | 0.5  (-1.7 to 2.4) | .84 |
| Week 14 | 44.1  (42.8 to 45.3) | 44.6  (43.3 to 45.8) | 44.3  (43.0 to 45.5) | 8.4  (7.3 to 9.5) | <.001 | 8.7  (7.6 to 9.9) | <.001 | 7.8  (6.6 to 8.9) | <.001 | -0.2  (-1.7 to 2.4) | .95 | 0.3  (-1.5 to 2.6) | .93 |
| Week 26 | 45.0  (43.7 to 46.2) | 45.2  (44.0 to 46.4) | 44.7  (43.5 to 45.9) | 9.3  (8.2 to 10.4) | <.001 | 9.4  (8.2 to 10.6) | <.001 | 8.2  (7.0 to 9.3) | <.001 | 0.3  (-1.5 to 2.6) | .96 | 0.5  (-1.7 to 2.4) | .80 |
| **SF-12 MCS^c^** | | | | | | | | | | | | | |
| Day 0 | 45.7  (44.3 to 47.1) | 44.96  (43.6 to 46.3) | 46.4  (45.1 to 47.7) |  |  |  |  |  |  | -0.7  (-3.6 to 0.7) | .74 | -1.4  (-3.0 to 1.3) | .24 |
| Day 3 | 46.1  (44.8 to 47.4) | 46.1  (44.8 to 47.4) | 47.3  (46.0 to 48.6) | 0.4  (-0.7 to 1.5) | .47 | 1.1  (0.0 to 2.2) | .049 | 0.9  (-0.2 to 2.0) | .10 | -1.2  (-3.4 to 0.9) | .38 | -1.2  (-2.2 to 2.1) | .34 |
| Week 1 | 47.9  (46.6 to 49.1) | 47.6  (46.3 to 48.9) | 47.8  (46.5 to 49.0) | 2.1  (1.1 to 3.2) | <.001 | 2.6  (1.5 to 3.8) | <.001 | 1.4  (0.3 to 2.5) | .01 | 0.1  (-2.4 to 1.9) | .99 | -0.2  (-2.6 to 1.8) | .97 |
| Week 2 | 48.7  (47.4 to 50.0) | 47.9  (46.6 to 49.1) | 49.2  (47.9 to 50.4) | 3.0  (1.9 to 4.1) | <.001 | 2.9  (1.8 to 4.0) | <.001 | 2.8  (1.7 to 3.9) | <.001 | -0.4 (-3.5 to 0.8) | .87 | -1.3  (-3.1 to 1.2) | .28 |
| Week 6 | 50.0  (48.9 to 51.2) | 48.8  (47.5 to 50.1) | 49.5  (48.3 to 50.7) | 4.3  (3.2 to 5.4) | <.001 | 3.8  (2.7 to 4.9) | <.001 | 3.1  (2.0 to 4.2) | <.001 | 0.5  (-2.9 to 1.4) | .84 | -0.7  (-3.5 to 0.8) | .66 |
| Week 14 | 50.3  (49.2 to 51.5) | 49.4  (48.1 to 50.7) | 50.4  (49.2 to 51.5) | 4.6  (3.5 to 5.7) | <.001 | 4.4  (3.3 to 5.5) | <.001 | 4.0  (2.9 to 5.1) | <.001 | 0.0  (-3.2 to 1.1) | .99 | -1.0  (-3.2 to 1.1) | .49 |
| Week 26 | 51.2  (50.0 to 52.4) | 50.1  (48.8 to 51.4) | 51.0  (49.8 to 52.2) | 5.5  (4.4 to 6.5) | <.001 | 5.1  (4.0 to 6.3) | <.001 | 4.6  (3.5 to 5.7) | <.001 | 0.2  (-3.1 to 1.2) | .98 | -0.9  (-3.2 to 1.1) | .54 |

Abbreviations: CI, confidence interval; LOPM, lumbar oblique pull manipulation; MCS, Mental Component Summary; ODI, Oswestry Disability Index; PCS, Physical Component Summary; YBT, Yaobitong; SF-12, 12-Item Short Form Health Survey; VAS, visual analog scale.

^a^ ODI assesses the effect of pain on normal daily activity including the ability to and intensity of lifting, care for oneself, walk, sit, sexual function, stand, social life, sleep and travel, ranging from 0 (no disability) to 100 (maximum disability possible).

^b^ Scores for leg pain and low back pain could range from 0 (no pain) to 10 (pain as bad as you can imagine).

^c^ SF-12 assesses the quality of life through physical and mental dimensions, with scores typically ranging from 0 to 100. Higher scores indicate a better quality of life.


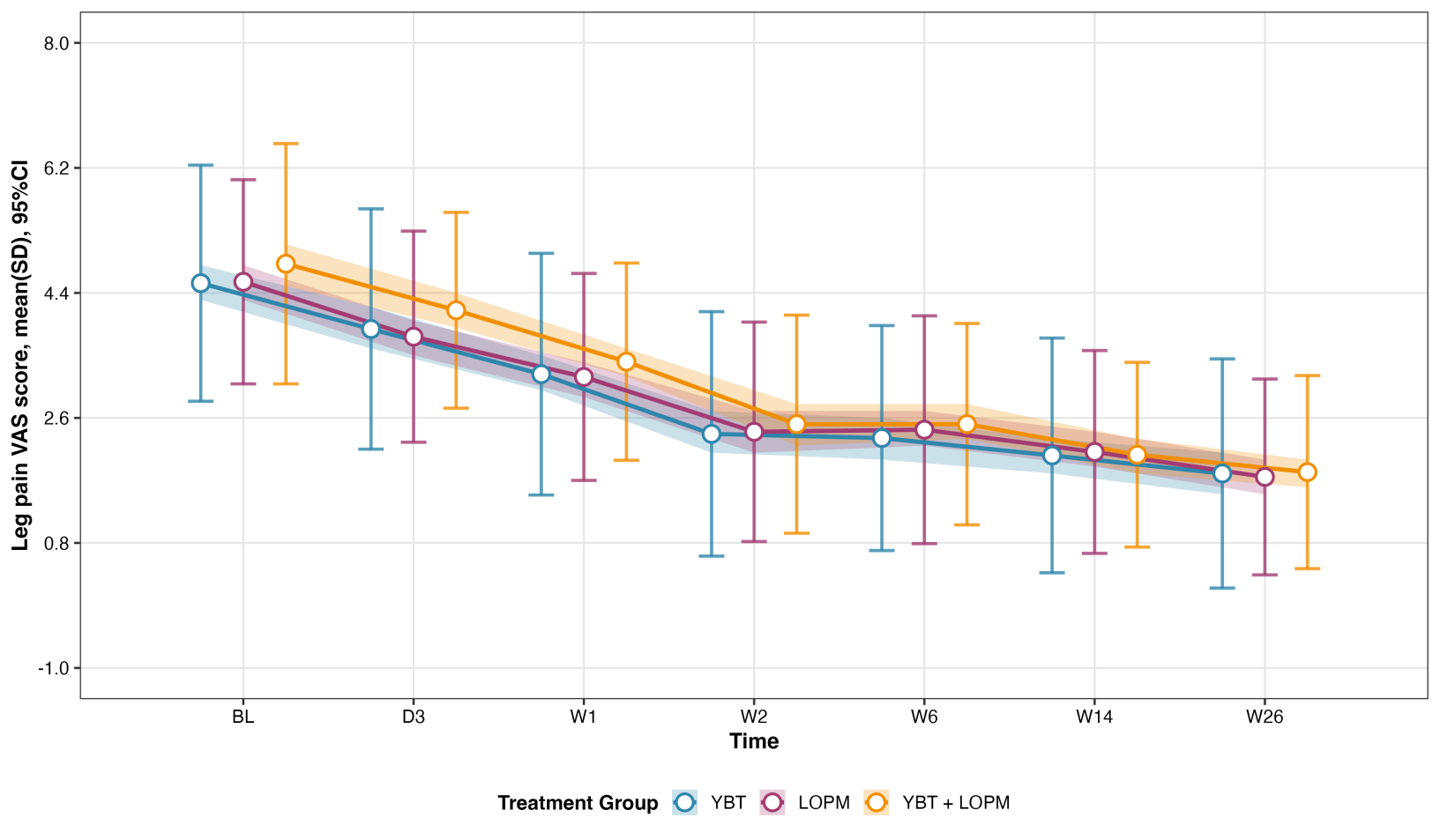


**eFigure 1. Trajectory of Leg Pain VAS Scores Over Time.** Abbreviations: BL, baseline; CI, confidence interval; D3, day 3; LOPM, lumbar oblique pull manipulation; SD, standard deviation; VAS, visual analog scale; W1, week 1; W2, week 2; W6, week 6; W14, week 14; W26, week 26; YBT, Yaobitong.


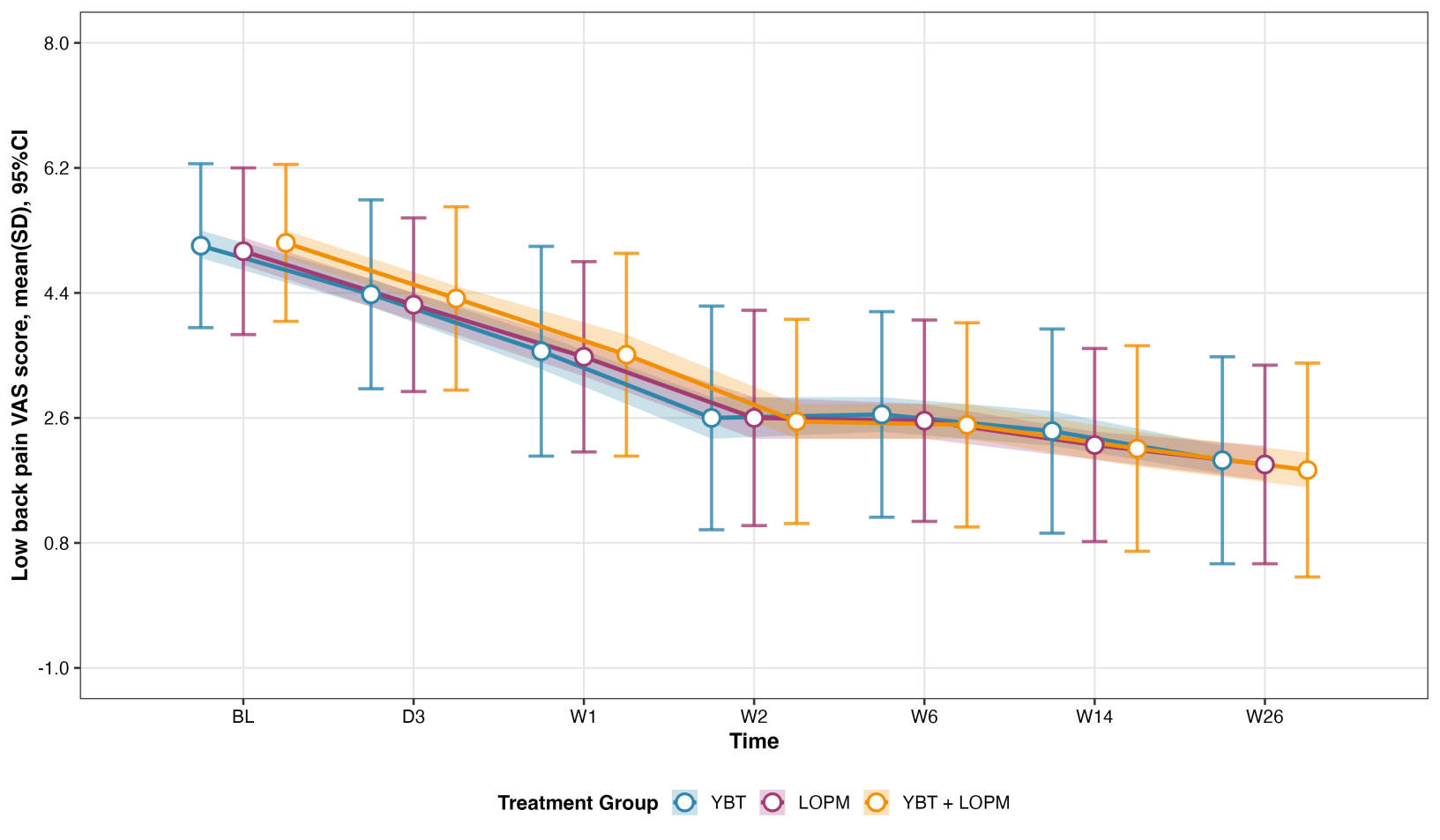


**eFigure 2. Trajectory of Low Back Pain VAS Scores Over Time.** Abbreviations: BL, baseline; CI, confidence interval; D3, day 3; LOPM, lumbar oblique pull manipulation; SD, standard deviation; VAS, visual analog scale; W1, week 1; W2, week 2; W6, week 6; W14, week 14; W26, week 26; YBT, Yaobitong.

**
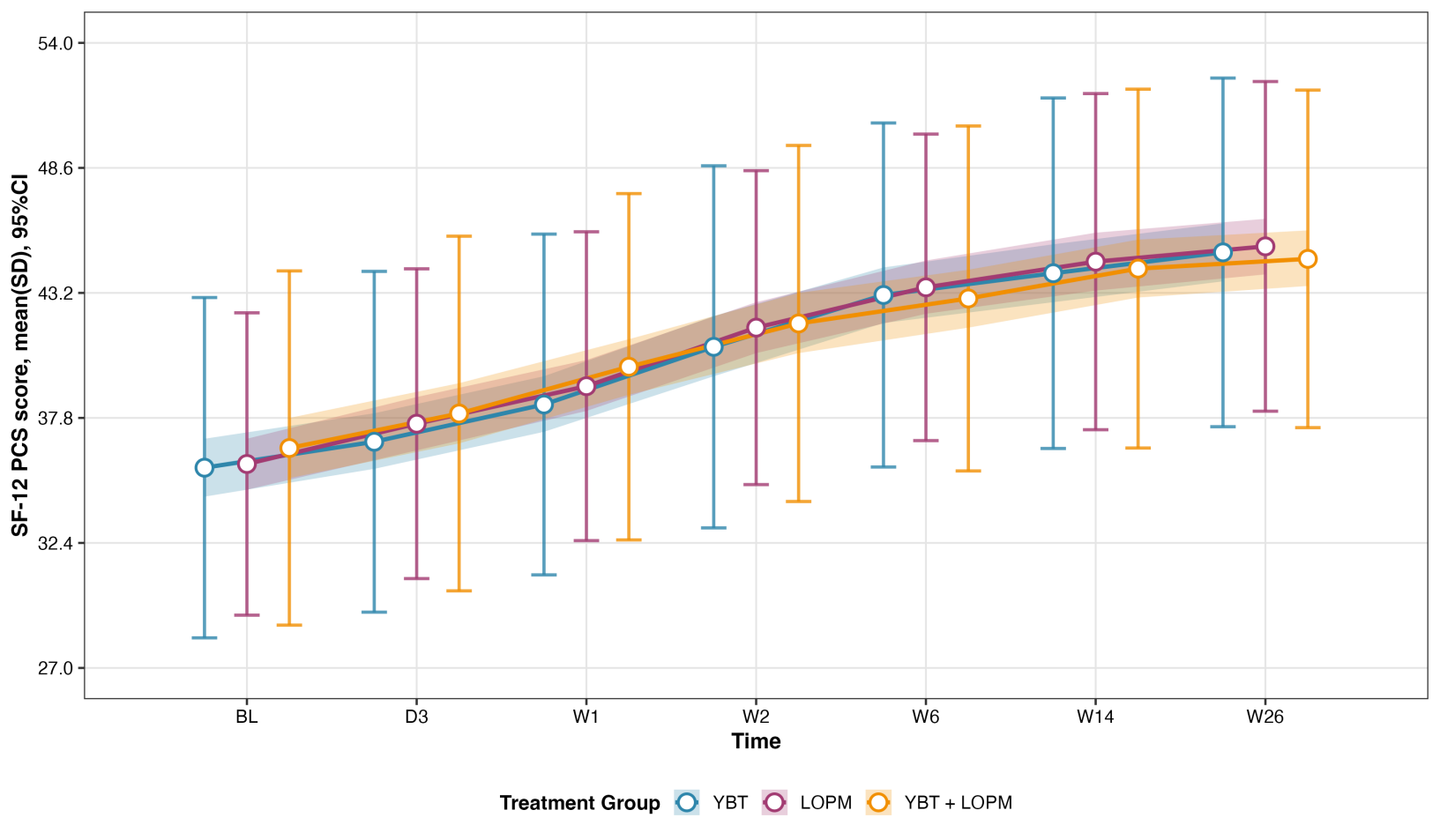
**

**eFigure 3. Trajectory of SF-12 PCS Scores Over Time.** Abbreviations: BL, baseline; CI, confidence interval; D3, day 3; LOPM, lumbar oblique pull manipulation; PCS, Physical Component Summary; SD, standard deviation; SF-12, 12-Item Short Form Health Survey; W1, week 1; W2, week 2; W6, week 6; W14, week 14; W26, week 26; YBT, Yaobitong.

**
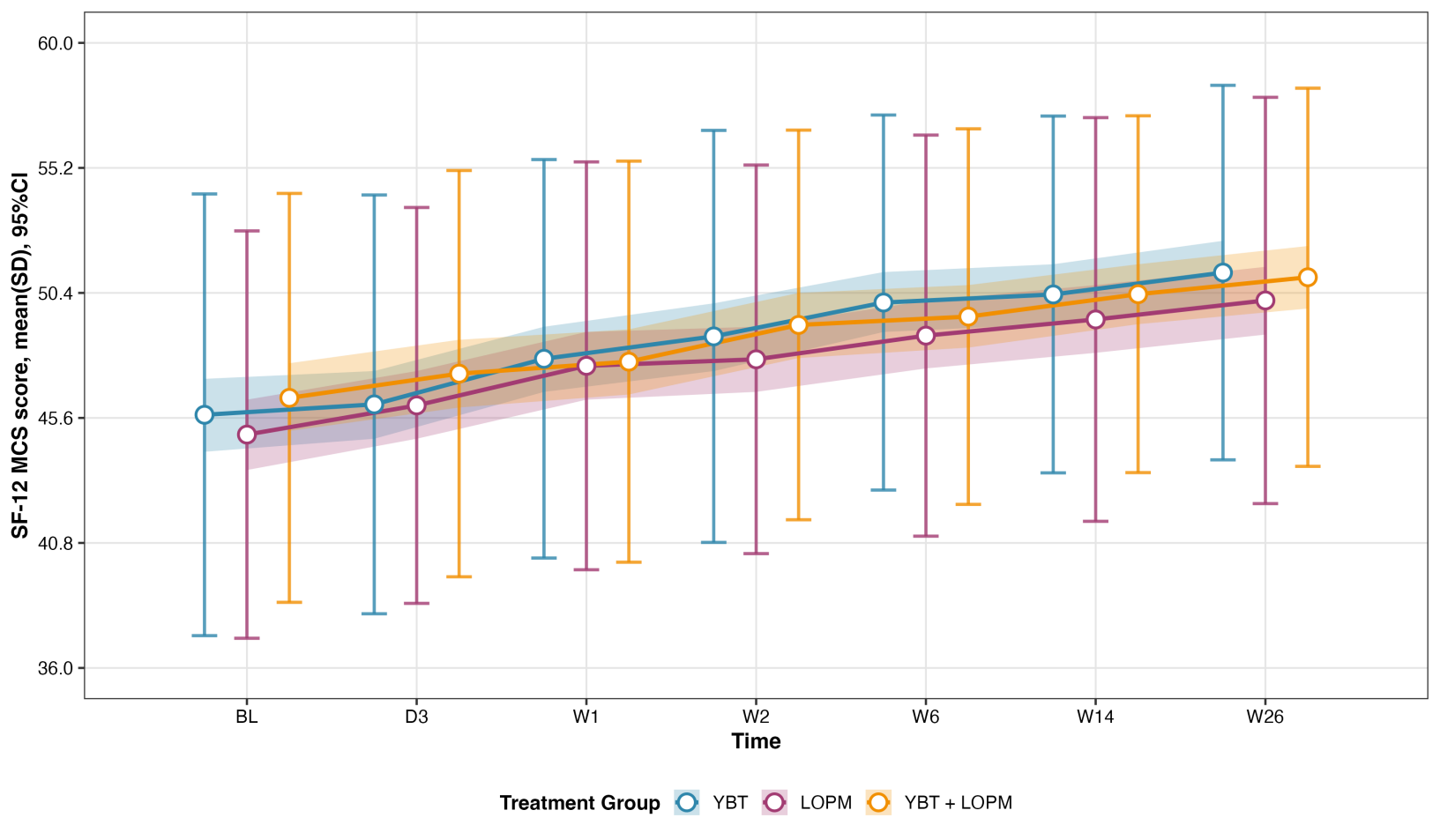
**

**eFigure 4. Trajectory of SF-12 MCS Scores Over Time.** Abbreviations: BL, baseline; CI, confidence interval; D3, day 3; LOPM, lumbar oblique pull manipulation; MCS, Mental Component Summary; SD, standard deviation; SF-12, 12-Item Short Form Health Survey; W1, week 1; W2, week 2; W6, week 6; W14, week 14; W26, week 26; YBT, Yaobitong.

**eTable 3. Outcome Measures in the Per-protocol Population**

|  | **Mean (95%CI)** | | | **Changes from baseline to each observation time point，mean (95%CI) / *p* value** | | | | | | **Difference between arms，mean (95%CI) / p value** | | | |
| --- | --- | --- | --- | --- | --- | --- | --- | --- | --- | --- | --- | --- | --- |
| **Follow up time** | **YBT**  **(n=145)** | **LOPM**  **(n=136)** | **YBT + LOPM**  **(n=142)** | **YBT** | | **LOPM** | | **YBT + LOPM** | | **YBT and YBT + LOPM** | | **LOPM and YBT + LOPM** | |
| **ODI^a^** | | | | | | | | | | | | | |
| Day 0 | 37.5  (35.0 to 40.0) | 38.5  (35.8 to 41.2) | 38.8  (36.1 to 41.5) |  |  |  |  |  |  | -1.4  (-1.5 to 5.7) | .36 | -0.3  (-3.9 to 3.3) | .98 |
| Day 3 | 30.6  (28.3 to 32.9) | 31.4  (28.9 to 33.9) | 30.2  (27.4 to 33.0) | -6.9  (-8.8 to -5.0) | <.001 | -7.1  (-9.1 to -5.2) | <.001 | -8.6  (-10.5 to -6.7) | <.001 | 0.4  (-3.3 to 3.9) | .98 | 1.2  (-2.4 to 4.8) | .71 |
| Week 1 | 25.2  (23.0 to 27.3) | 26.1  (23.8 to 28.4) | 24.3  (22.0 to 26.6) | -12.3  (-14.2 to -10.4) | <.001 | -12.4  (-14.4 to -10.4) | <.001 | -14.5  (-16.4 to -12.6) | <.001 | 0.9  (-3.7 to 3.4) | .99 | 1.8  (-1.8 to 5.5) | .45 |
| Week 2 | 18.2  (16.2 to 20.1) | 19.0  (16.9 to 21.2) | 17.8  (15.7 to 19.9) | -19.3  (-21.2 to -17.4) | <.001 | -19.5  (-21.4 to -17.5) | <.001 | -21.0  (-23.0 to -19.1) | <.001 | 0.4  (-3.2 to 3.9) | .97 | 1.3  (-2.3 to 4.9) | .68 |
| Week 6 | 17.5  (15.6 to 19.5) | 17.8  (15.9 to 19.6) | 16.5  (14.6 to 18.4) | -19.9  (-21.8 to -18.0) | <.001 | -20.7  (-22.7 to -18.8) | <.001 | -22.4  (-24.3 to -20.4) | <.001 | 1.1  (-3.9 to 3.2) | .97 | 1.3  (-2.3 to 5.0) | .65 |
| Week 14 | 16.0  (13.9 to 18.0) | 16.3  (14.5 to 18.2) | 15.3  (13.5 to 17.1) | -21.5  (-23.4 to -19.6) | <.001 | -22.2  (-24.2 to -20.2) | <.001 | -23.5  (-25.4 to -21.6) | <.001 | 0.7  (-3.5 to 3.7) | .99 | 1.0  (-2.6 to 4.7) | .77 |
| Week 26 | 13.0  (11.3 to 14.6) | 13.2  (11.5 to 14.9) | 12.3  (10.5 to 14.0) | -24.5  (-26.4 to -22.6) | <.001 | -25.3  (-27.2 to -23.3) | <.001 | -26.5  (-28.4 to -24.6) | <.001 | 0.7  (-3.5 to 3.6) | .99 | 0.9  (-2.6 to 4.6) | .80 |
| **Leg pain VAS score^b^** | | | | | | | | | | | | | |
| Day 0 | 4.5  (4.3 to 4.8) | 4.6  (4.3 to 4.8) | 4.8  (4.6 to 5.1) |  |  |  |  |  |  | -0.3  (-0.8 to 0.1) | .14 | -0.3  (-0.7 to 0.2) | .32 |
| Day 3 | 3.9  (3.6 to 4.2) | 3.8  (3.5 to 4.0) | 4.1  (3.9 to 4.3) | -0.7  (-0.9 to -0.4) | <.001 | -0.8  (-1.0 to -0.6) | <.001 | -0.7  (-1.0 to -0.5) | <.001 | -0.2  (-0.7 to 0.2) | .31 | -0.4  (-0.8 to 0.1) | .19 |
| Week 1 | 3.2  (2.9 to 3.5) | 3.2  (2.9 to 3.4) | 3.4  (3.1 to 3.6) | -1.3  (-1.5 to -1.1) | <.001 | -1.4  (-1.6 to -1.2) | <.001 | -1.5  (-1.7 to -1.3) | <.001 | -0.1  (-0.3 to 0.6) | .57 | -0.2  (-0.6 to 0.3) | .60 |
| Week 2 | 2.4  (2.1 to 2.7) | 2.4  (2.1 to 2.7) | 2.5  (2.3 to 2.8) | -2.2  (-2.4 to -1.9) | <.001 | -2.2  (-2.4 to -1.9) | <.001 | -2.3  (-2.5 to -2.1) | <.001 | -0.1  (-0.3 to 0.6) | .55 | -0.1  (-0.6 to 0.3) | .83 |
| Week 6 | 2.3  (2.0 to 2.6) | 2.4  (2.1 to 2.7) | 2.5  (2.2 to 2.7) | -2.2  (-2.5 to -2.0) | <.001 | -2.2  (-2.4 to -1.9) | <.001 | -2.4  (-2.6 to -2.1) | <.001 | -0.2  (-0.2 to 0.7) | .40 | -0.1  (-0.5 to 0.4) | .93 |
| Week 14 | 2.1  (1.8 to 2.3) | 2.1  (1.9 to 2.4) | 2.1  (1.8 to 2.3) | -2.5  (-2.7 to -2.3) | <.001 | -2.5  (-2.7 to -2.2) | <.001 | -2.8  (-3.0 to -2.6) | <.001 | 0.0  (-0.4 to 0.5) | .95 | 0.1  (-0.4 to 0.5) | .95 |
| Week 26 | 1.8  (1.5 to 2.1) | 1.8  (1.5 to 2.0) | 1.8  (1.6 to 2.0) | -2.8  (-3.0 to -2.5) | <.001 | -2.8  (-3.0 to -2.6) | <.001 | -3.0  (-3.3 to -2.8) | <.001 | 0.0  (-0.4 to 0.5) | .93 | -0.0  (-0.5 to 0.4) | .99 |
| **Low back pain VAS score^b^** | | | | | | | | | | | | | |
| Day 0 | 5.1  (4.9 to 5.3) | 5.0  (4.8 to 5.2) | 5.1  (4.9 to 5.3) |  |  |  |  |  |  | -0.0  (-0.3 to 0.5) | .94 | -0.1  (-0.5 to 0.3) | .77 |
| Day 3 | 4.4  (4.2 to 4.6) | 4.2  (4.0 to 4.4) | 4.2  (4.0 to 4.5) | -0.7  (-0.9 to -0.5) | <.001 | -0.8  (-1.0 to -0.6) | <.001 | -0.9  (-1.1 to -0.7) | <.001 | 0.2  (-0.5 to 0.3) | .68 | -0.1  (-0.5 to 0.3) | .93 |
| Week 1 | 3.6  (3.3 to 3.8) | 3.5  (3.2 to 3.7) | 3.5  (3.2 to 3.7) | -1.5  (-1.7 to -1.3) | <.001 | -1.5  (-1.7 to -1.3) | <.001 | -1.7  (-1.9 to -1.5) | <.001 | 0.1  (-0.5 to 0.3) | .80 | 0.0  (-0.4 to 0.4) | .99 |
| Week 2 | 2.6  (2.4 to 2.9) | 2.6  (2.3 to 2.9) | 2.5  (2.3 to 2.8) | -2.4  (-2.7 to -2.2) | <.001 | -2.4  (-2.6 to -2.2) | <.001 | -2.6  (-2.8 to -2.4) | <.001 | 0.1  (-0.5 to 0.3) | .83 | 0.1  (-0.3 to 0.5) | .90 |
| Week 6 | 2.7  (2.4 to 2.9) | 2.5  (2.3 to 2.8) | 2.5  (2.2 to 2.7) | -2.4  (-2.6 to -2.2) | <.001 | -2.5  (-2.7 to -2.3) | <.001 | -2.7  (-2.9 to -2.4) | <.001 | 0.2  (-0.2 to 0.6) | .45 | 0.1  (-0.3 to 0.5) | .93 |
| Week 14 | 2.4  (2.2 to 2.7) | 2.2  (1.9 to 2.4) | 2.1  (1.9 to 2.3) | -2.6  (-2.8 to -2.4) | <.001 | -2.8  (-3.0 to -2.6) | <.001 | -3.0  (-3.2 to -2.8) | <.001 | 0.3  (-0.1 to 0.7) | .16 | 0.1  (-0.3 to 0.5) | .94 |
| Week 26 | 2.0  (1.8 to 2.3) | 1.9  (1.7 to 2.2) | 1.8  (1.6 to 2.1) | -3.1  (-3.3 to -2.9) | <.001 | -3.1  (-3.3 to -2.9) | <.001 | -3.3  (-3.5 to -3.1) | <.001 | 0.2  (-0.6 to 0.2) | .55 | 0.1  (-0.3 to 0.5) | .84 |
| **SF-12 PCS^c^** | | | | | | | | | | | | | |
| Day 0 | 35.6  (34.4 to 36.8) | 35.8  (34.7 to 36.9) | 36.5  (35.2 to 37.8) |  |  |  |  |  |  | -0.9  (-1.1 to 3.0) | .52 | -0.7  (-2.7 to 1.4) | .72 |
| Day 3 | 36.7  (35.5 to 37.9) | 37.6  (36.4 to 38.7) | 37.9  (36.7 to 39.2) | 1.0  (-0.1 to 2.2) | .07 | 1.7  (0.6 to 2.9) | .004 | 1.4  (0.3 to 2.5) | .02 | -1.3  (-3.3 to 0.7) | .29 | -0.4  (-2.4 to 1.7) | .91 |
| Week 1 | 38.4  (37.2 to 39.6) | 39.2  (38.1 to 40.3) | 40.0  (38.7 to 41.2) | 2.8  (1.6 to 3.9) | <.001 | 3.4  (2.2 to 4.5) | <.001 | 3.4  (2.3 to 4.6) | <.001 | -1.6  (-3.7 to 0.4) | .14 | -0.8  (-2.8 to 1.3) | .65 |
| Week 2 | 40.8  (39.6 to 42.1) | 41.7  (40.5 to 42.8) | 42.0  (40.7 to 43.2) | 5.2  (4.1 to 6.3) | <.001 | 5.9  (4.7 to 7.0) | <.001 | 5.5  (4.3 to 6.6) | <.001 | -1.1  (-3.2 to 0.8) | .35 | -0.3  (-2.4 to 1.8) | .94 |
| Week 6 | 43.2  (42.0 to 44.4) | 43.5  (42.3 to 44.6) | 43.0  (41.7 to 44.2) | 7.5  (6.4 to 8.7) | <.001 | 7.6  (6.5 to 8.8) | <.001 | 6.5  (5.3 to 7.6) | <.001 | 0.2  (-2.2 to 1.9) | .99 | 0.5  (-1.6 to 2.5) | .85 |
| Week 14 | 44.0  (42.8 to 45.3) | 44.6  (43.4 to 45.8) | 44.3  (43.0 to 45.6) | 8.4  (7.3 to 9.5) | <.001 | 8.8  (7.6 to 9.9) | <.001 | 7.8  (6.6 to 8.9) | <.001 | -0.3  (-1.7 to 2.3) | .93 | 0.3  (-1.7 to 2.4) | .93 |
| Week 26 | 44.9  (43.7 to 46.2) | 45.3  (44.1 to 46.5) | 44.6  (43.4 to 45.8) | 9.3  (8.2 to 10.4) | <.001 | 9.5  (8.3 to 10.6) | <.001 | 8.1  (7.0 to 9.2) | <.001 | 0.3  (-2.3 to 1.8) | .95 | 0.7  (-1.4 to 2.8) | .70 |
| **SF-12 MCS^c^** | | | | | | | | | | | | | |
| Day 0 | 45.7  (44.3 to 47.1) | 44.9  (43.6 to 46.2) | 46.3  (45.0 to 47.6) |  |  |  |  |  |  | -0.7  (-1.4 to 2.8) | .71 | -1.4  (-3.7 to 0.6) | .22 |
| Day 3 | 46.1  (44.7 to 47.4) | 46.0  (44.8 to 47.3) | 47.3  (46.0 to 48.6) | 0.4  (-0.7 to 1.5) | .49 | 1.1  (0.0 to 2.3) | .045 | 1.0  (-0.1 to 2.1) | .08 | -1.3  (-0.8 to 3.5) | .32 | -1.3  (-3.5 to 0.8) | .30 |
| Week 1 | 47.8  (46.6 to 49.1) | 47.6  (46.3 to 48.9) | 47.7  (46.4 to 49.0) | 2.2  (1.1 to 3.3) | <.001 | 2.7  (1.6 to 3.8) | <.001 | 1.4  (0.3 to 2.5) | .01 | 0.1  (-2.2 to 2.1) | .99 | -0.1  (-2.4 to 2.0) | .97 |
| Week 2 | 48.7  (47.4 to 50.0) | 47.9  (46.6 to 49.1) | 49.1  (47.9 to 50.3) | 3.0  (1.9 to 4.1) | <.001 | 2.9  (1.8 to 4.1) | <.001 | 2.8  (1.7 to 3.9) | <.001 | -0.4  (-1.7 to 2.6) | .86 | -1.3  (-3.5 to 0.8) | .32 |
| Week 6 | 50.1  (48.9 to 51.3) | 48.7  (47.4 to 50.0) | 49.5  (48.3 to 50.7) | 4.4  (3.3 to 5.5) | <.001 | 3.8  (2.7 to 4.9) | <.001 | 3.1  (2.0 to 4.2) | <.001 | 0.6  (-2.7 to 1.5) | .79 | -0.8  (-3.0 to 1.3) | .63 |
| Week 14 | 50.3  (49.2 to 51.4) | 49.3  (48.0 to 50.6) | 50.3  (49.2 to 51.5) | 4.6  (3.6 to 5.7) | <.001 | 4.4  (3.3 to 5.5) | <.001 | 4.0  (2.9 to 5.1) | <.001 | -0.0  (-2.1 to 2.2) | .99 | -1.0  (-3.3 to 1.1) | .46 |
| Week 26 | 51.1  (50.0 to 52.3) | 50.1  (48.8 to 51.5) | 51.0  (49.8 to 52.2) | 5.5  (4.4 to 6.6) | <.001 | 5.2  (4.1 to 6.3) | <.001 | 4.6  (3.5 to 5.7) | <.001 | 0.2  (-2.3 to 2.0) | .99 | -0.9  (-3.1 to 1.2) | .57 |

Abbreviations: CI, Confidence interval; LOPM, lumbar oblique pull manipulation; MCS, Mental Component Summary; ODI, Oswestry Disability Index; PCS, Physical Component Summary; YBT, Yaobitong; SF-12, 12-Item Short Form Health Survey; VAS, visual analog scale.

^a^ ODI assesses the effect of pain on normal daily activity including the ability to and intensity of lifting, care for oneself, walk, sit, sexual function, stand, social life, sleep and travel, ranging from 0 (no disability) to 100 (maximum disability possible).

^b^ Scores for leg pain and low back pain could range from 0 (no pain) to 10 (pain as bad as you can imagine).

^c^ SF-12 assesses the quality of life through physical and mental dimensions, with scores typically ranging from 0 to 100. Higher scores indicate a better quality of life.

**eTable 4. Proportion of Patients Achieving the Minimal Clinically Important Difference (≥7-Point Oswestry Disability Index Reduction) at Week 2 in the Per-Protocol Population**

| **No. (%) (95% CI)** | | | **Difference between groups, p value** | |
| --- | --- | --- | --- | --- |
| **YBT (n=145)** | **LOPM (n=136)** | **YBT + LOPM (n=142)** | **YBT and YBT + LOPM** | **LOPM and YBT + LOPM** |
| 121 (83.4) (76.2, 88.9) | 110 (80.9) (73.1, 86.9) | 123 (86.6) (79.6, 91.5) | .45 | .19 |

Abbreviations: CI, Confidence interval; LOPM, lumbar oblique pull manipulation; YBT, Yaobitong.

**eTable 5. Adverse Events**

| **Adverse event** | **Group** | **Timing (day)** | **Severity** | **Serious** | **Relationship to treatment** | **Management** | **Outcome** |
| --- | --- | --- | --- | --- | --- | --- | --- |
| Skin allergy | YBT + LOPM | Day 4 | Mild | No | Possible | None | Resolved in 3 days |
| Abdominal pain with diarrhea | YBT + LOPM | Day 2 | Mild | No | Possible | None | Resolved in 2 days |

Abbreviations: LOPM, lumbar oblique pull manipulation; YBT, Yaobitong

**eTable 6. Reasons for Missing 26-week MRI Data**

|  | **No. (%)** | | | |
| --- | --- | --- | --- | --- |
|  | **YBT (n=146)** | **LOPM (n=137)** | **YBT + LOPM (n=143)** | **Total (N=426)** |
| Refusal of repeat MRI | 35 (24.0) | 33 (24.1) | 35 (24.5) | 103 (24.2) |
| Scheduling conflicts | 14 (9.6) | 13 (9.5) | 11 (7.7) | 38 (8.9) |
| Withdrew from trial | 0 (0) | 1 (0.7) | 0 (0) | 1 (0.2) |
| Lost to follow-up | 1 (0.7) | 0 (0) | 1 (0.7) | 2 (0.5) |
| Total missing | 50 (34.2) | 47 (34.3) | 47 (32.9) | 144 (33.8) |

Abbreviations: LOPM, lumbar oblique pull manipulation; YBT, Yaobitong.
